# A Comprehensive Lateral Flow Strip Assay for On‐Site mRNA Vaccine Quality Control in Decentralized Manufacturing

**DOI:** 10.1002/advs.202502387

**Published:** 2025-09-03

**Authors:** Dengwang Luo, Ziwei Zhang, Jing Guo, Jieli Zhang, Arong Huang, Qinghao Cao, Jiangnan Zheng, Yingjin Yuan, Daming Wang, Yuhong Cao

**Affiliations:** ^1^ State Key Laboratory of Synthetic Biology Frontiers Science Center for Synthetic Biology (Ministry of Education) School of Synthetic Biology and Biomanufacturing Tianjin University Tianjin 300072 China; ^2^ CAS Key Laboratory for Biological Effects of Nanomaterials and Nanosafety National Center for Nanoscience and Technology Chinese Academy of Sciences Beijing 100190 China; ^3^ College of Nanoscience and Technology University of Chinese Academy of Sciences Beijing 100049 China; ^4^ Anbio Biotechnology Company Xiamen Fujian 361026 China; ^5^ CAS Key Laboratory of Molecular Recognition and Function Chinese Academy of Sciences Beijing 100190 China; ^6^ BiosynRNA Biotechnology Company Beijing 100192 China

**Keywords:** mRNA vaccine, lateral flow strip assay, 5' capping efficiency, integrity, encapsulation efficiency

## Abstract

The rapid adoption of mRNA‐based vaccines highlights the critical need for on‐site quality control (QC) methods, particularly in low‐income countries with decentralized manufacturing. Existing techniques, such as liquid chromatography‐mass spectrometry (LC‐MS) and capillary electrophoresis (CE), are resource‐intensive, requiring specialized equipment and expertise. To address this, a comprehensive lateral flow strip assay (LFSA) has been developed to evaluate key mRNA quality attributes—5' capping efficiency, integrity, and lipid nanoparticles (LNPs) encapsulation efficiency. Leveraging antibody‐labeled fluorescence nanoparticles and polydeoxythymidine oligonucleotide to simultaneously probe both 5' cap and poly(A) tail, the LFSA offers sequence‐independent, rapid (15 min), and sensitive mRNA analysis. Validation against standard methods demonstrates comparable accuracy while significantly reducing sample requirements and operational complexity. Moreover, the LFSA enables real‐time monitoring of mRNA stability under varying storage conditions and provides precise encapsulation assessments, distinguishing between intact and degraded mRNA in LNPs. This portable and cost‐effective platform bridges critical gaps in mRNA QC, streamlines manufacturing workflows, and ensures mRNA therapeutic efficacy in diverse applications.

## Graphical abstract

A rapid, portable lateral flow strip assay for on‐site mRNA quality control is developed, enabling sequence‐independent analysis of 5' capping, integrity, and LNPs encapsulation efficiency. This cost‐effective method offers real‐time stability monitoring, reduced sample requirements, and comparable accuracy to standard techniques, enhancing manufacturing workflows and ensuring mRNA therapeutic efficacy in decentralized settings.

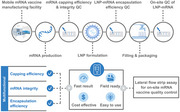

## 1 Introduction

The rapid development of mRNA‐based vaccines during the COVID‐19 pandemic has underscored the immense potential of mRNA technology to provide swift responses to emerging infectious diseases.^[^ ^1^ ^]^ mRNA vaccines offer significant advantages, including rapid design and scalability, making them essential tools in addressing global health emergencies.^[^ ^2^ ^]^ However, a major challenge lies in the inherent sensitivity of mRNA to temperature fluctuations, which necessitates strict cold‐chain transportation and storage conditions.^[^ ^3^ ^]^ This requirement imposes substantial logistical hurdles, particularly in low‐income countries with limited infrastructure, thereby restricting equitable access to these lifesaving therapeutics.^[^ ^4^ ^]^

To mitigate these challenges, the establishment of miniaturized, local manufacturing platforms has emerged as a promising solution. Leveraging the modular nature of mRNA production, BioNTech introduced containerized mRNA manufacturing units in 2022.^[^ ^5^ ^]^ These mobile facilities can be rapidly deployed in diverse locations, enabling on‐site production of mRNA vaccines. This innovation has the potential to significantly improve global health equity by facilitating localized vaccine production, reducing reliance on complex supply chains, and overcoming the limitation of cold‐chain storage.

Despite these advances, ensuring consistent QC of mRNA products remains a complex and resource‐intensive process.^[^ ^6^ ^]^ Critical quality attributes, including mRNA capping efficiency, integrity, and lipid nanoparticles (LNPs) encapsulation efficiency, are typically assessed using sophisticated techniques such as LC‐MS, CE, and the Ribogreen assay.^[^ ^7^ ^]^ Specifically, LC‐MS is costly and time‐consuming, requiring specialized personnel and laboratory infrastructure, while CE, though cheaper, still demands expertise and high sample costs. The Ribogreen assay is simple and sensitive for total RNA quantification, but it cannot distinguish between intact mRNA and partially degraded fragments. These methods must be performed in a laboratory setting, making them impractical for rapid, on‐site QC in decentralized manufacturing.

In contrast to these traditional methods, the LFSA presents a promising alternative for mRNA QC due to its speed and cost‐effectiveness.^[^ ^8^ ^]^ However, applying LFSA technology for mRNA QC faces significant technical challenges. Detecting specific mRNA structural features, ensuring sensitivity to distinguish intact mRNA from degraded forms, and accurate quantification have proven difficult.^[^ ^9^ ^]^ These challenges are further compounded by the need for precise measurements essential for therapeutic applications of mRNA.

**Figure 1:**
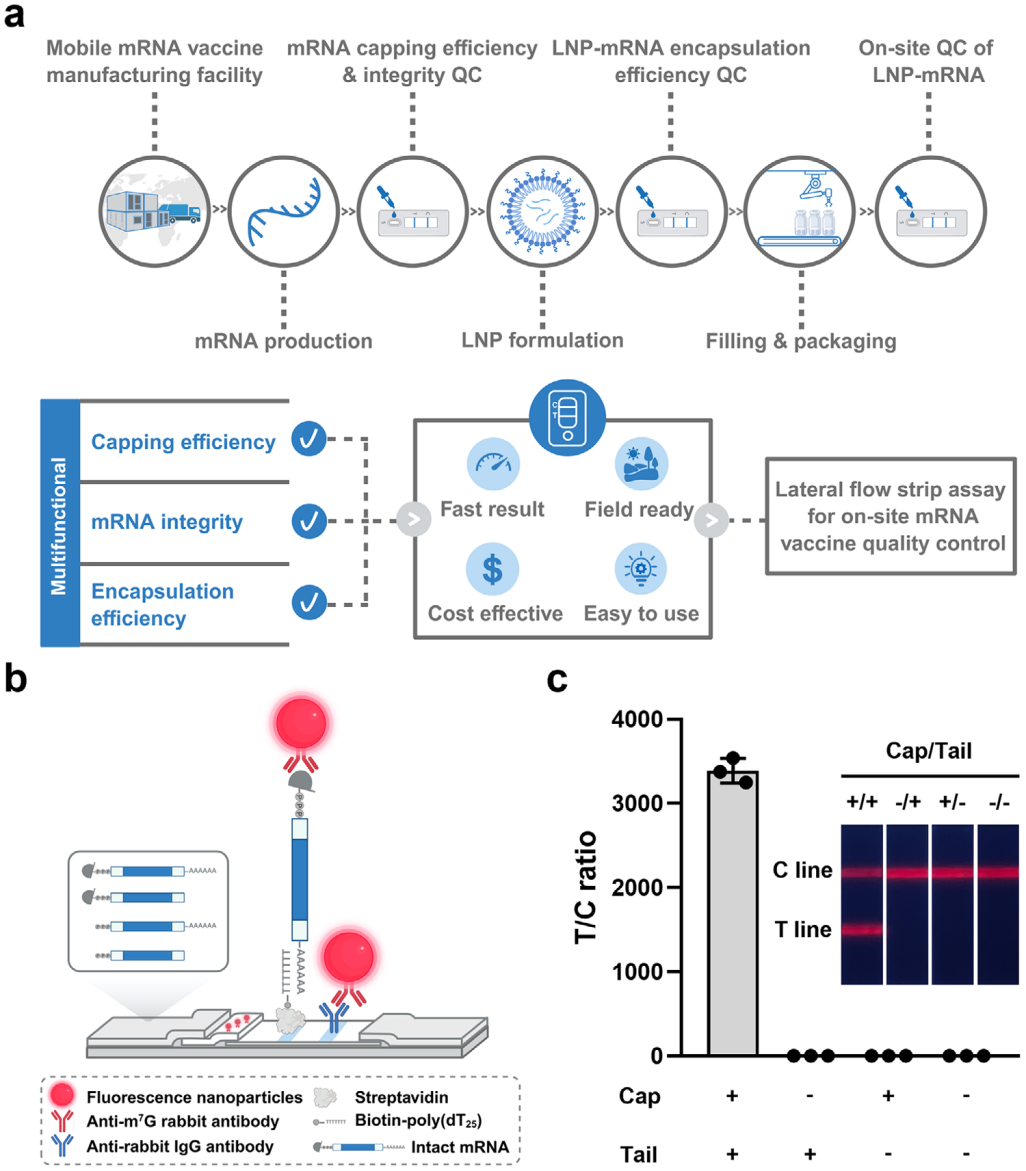
The LFSA‐mRNA detection system. a) Schematic illustration of a comprehensive lateral flow strip assay for on‐site quality control of mRNA vaccines in decentralized manufacturing settings. b) Working principle of the LFSA, featuring fluorescent signal outputs for detecting intact mRNA. c) Representative results of various Firefly luciferase (Fluc) mRNA samples tested on lateral flow strips, recorded using an immunofluorescence analyzer (Anbio, AF‐100S) and photographed under a UV lamp (*n* = 3). The ratio of the test fluorescence signal to the control fluorescence signal (T/C) on the lateral flow strip was used as a quantitative parameter to assess intact mRNA. Panels a and b were created with BioRender.com.

To address these barriers, we developed a novel LFSA for on‐site evaluation of key mRNA vaccine quality attributes. We integrated fluorescence nanoparticle probes and optimized the strip design to enhance specificity and sensitivity, enabling accurate quantitative analysis. The assay detects both the 5' cap and poly(A) tail, providing real‐time measurements of capping efficiency, integrity, and encapsulation efficiency in decentralized manufacturing. Importantly, it operates without the need for complex laboratory setups. (**Figure** **1**a).

## 2 Results

### 2.1 Design and Optimization of LFSA

To enable universal mRNA detection, we developed a fluorescence‐based assay using fluorescence nanoparticles (FNPs) conjugated with anti‐7‐methylguanosine (m^7^G) rabbit monoclonal antibodies as detection probes to specifically bind the 5' cap structure. For capturing mRNA, we employed poly(dT_25_) probes to hybridize with the poly(A) tail (Figure 1b). This dual‐probe strategy allows selective detection of fully intact mRNA molecules possessing both the 5' cap and poly(A) tail, resulting in a fluorescence signal upon formation of the FNP‐mRNA complex. In contrast, mRNA lacking these features produces no fluorescence signal at the T line (Figure 1c and Figure S1, Supporting Information). Additionally, this design mitigates heterophile interference, a common issue in conventional double‐antibody sandwich methods, thereby improving overall sensitivity and reducing background noise. The assay process, from sample loading to result acquisition, takes only 15 min, minimizing the risk of mRNA degradation during testing. Importantly, this approach is sequence‐independent, making the LFSA applicable to various mRNAs.

To further enhance the assay's specificity and accuracy, we optimized the buffer conditions. Adjustments to NP40, NaCl, and formamide concentrations were made to reduce false positive results and improve the signal‐to‐noise ratio (Figure S2, Supporting Information). The optimal buffer formulation, containing 0.2% NP40, 3% NaCl, and 2% formamide in 10 mM Tris‐HCl (pH 7.4), minimized nonspecific binding and ensured reliable detection of capped and tailed mRNA across different sequences.

### 2.2 The Influence of mRNA Structures on LFSA Signals

Compared to other biomolecules with defined structures, mRNA structure is highly versatile, influenced by multiple parameters such as length, chemical modification, capping chemistry, and sequence composition.^[^ ^10^ ^]^ These structural variations play a significant role in determining the signal intensity on the strip. Through our analysis, we observed that mRNAs with different structural properties produced varying signal intensities on the strips, reflecting the complexity of mRNA detection and quantification.

**Figure 2:**
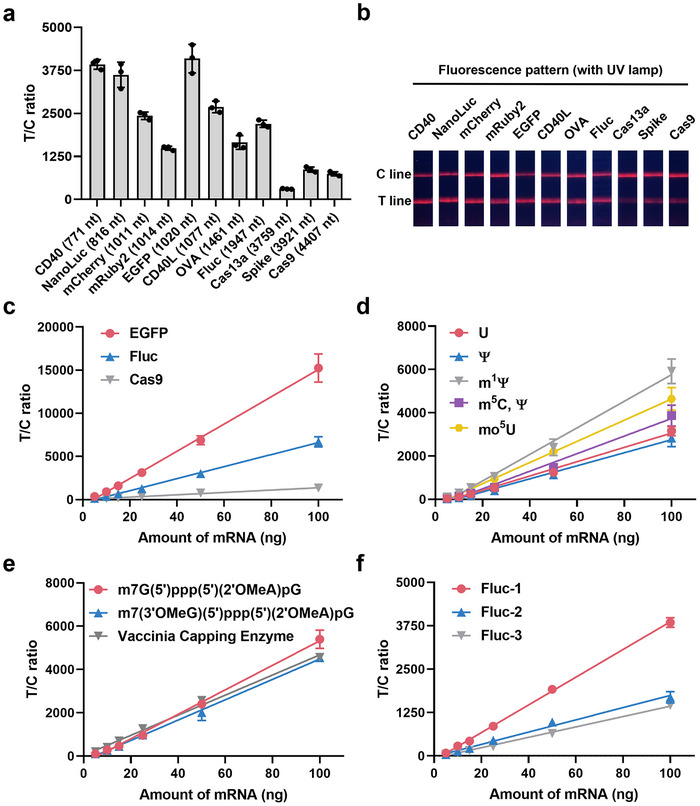
Test strip results for mRNAs with different structural features. a) T/C ratios of various mRNAs loaded onto lateral flow strips (*n *= 3). The mRNAs tested and their nucleotide lengths are: CD40 (771 nt), NanoLuc (816 nt), mCherry (1011 nt), mRuby2 (1014 nt), EGFP (1020 nt), CD40L (1077 nt), OVA (1461 nt), Fluc (1947 nt), Cas13a (3759 nt), Spike (3921 nt), and Cas9 (4407 nt). b) Photographs of the strips loaded with different mRNAs taken under a UV lamp. c‐f) Linear regression analysis of T/C ratios for: c) varying amounts of mRNAs (encoding EGFP, Fluc, and Cas9); d) Fluc mRNAs with different nucleotide modifications (U, Ψ, m^1^Ψ, m⁵C/Ψ, and mo⁵U); e) Fluc mRNAs synthesized with co‐transcriptional capping (two cap analog types) or post‐transcriptional capping (vaccinia capping enzyme); f) Fluc mRNAs with three coding sequences derived from BiosynRNA Biotechnology Co., Ltd. (*n* = 3).

To validate the LFSA's ability for mRNA quantification, we systematically evaluated the influence of mRNA structures by testing mRNAs of different lengths, chemical modifications, capping chemistries, and sequences encoding the same gene (Methods). Firstly, we tested mRNAs ranging from 771 to 4,407 nucleotides in length to confirm the LFSA's universality (**Figure** **2**a,b) and generated standard curves using EGFP, Fluc, and Cas9 mRNA sequences (Figure 2c) to evaluate the LFSA's quantification performance. These three transcripts were chosen because they are widely used representative mRNAs, cover a broad size range (≈1000 to >4000 nt), and are commonly applied in method validation, ensuring broad applicability and reproducibility. We noted that as the length of mRNA increased, the signal intensity on the LFSA decreased. This reduction in signal may be attributed to steric hindrance and the increased structural complexity of longer mRNAs, which could interfere with probe binding efficiency. Longer mRNAs likely adopt more intricate secondary and tertiary structures, which may obscure the 5' cap or poly(A) tail, making it more difficult for the detection probes to access these regions. Despite this variation in signal intensity, the LFSA demonstrated quantitative accuracy within 5%, indicating that mRNA length does not significantly interfere with the assay's ability to quantify mRNA amount (Figure 2c; Figures S3, and S4a, Supporting Information).

We further investigated the effects of different chemical modifications and capping chemistries on LFSA performance. The results suggested that neither parameter significantly influenced the quantitative capabilities of the LFSA. Interestingly, chemical modifications of nucleotides had a stronger effect on LFSA signal intensity than variations in capping chemistry (Figure 2d,e; Figure S4b,c, Supporting Information). This result was unexpected, since anti‐cap antibodies are typically highly sensitive to antigenic variations. Nonetheless, the LFSA proved more sensitive to chemical modifications, likely due to their greater influence on the overall mRNA structure. Chemical modifications of nucleotides can significantly alter the folding and stability of mRNA, affecting how the detection probes interact with the molecule.

To further investigate the influence of mRNA structure on LFSA performance, we examined different mRNA sequences encoding the same gene. Sequence variations notably affected signal intensity, likely by altering secondary structures that limit probe accessibility. (Figure 2f; Figure S4d, Supporting Information). Despite these sequence‐dependent differences in signal intensity, the LFSA consistently maintained quantitative accuracy within 5%. This underscores the robustness of the assay for quantitative analysis, even in the presence of structural variability due to sequence differences.

### 2.3 5' Capping Efficiency Analysis of mRNA by LFSA

The 5' cap structure of mRNA is crucial for efficient protein translation, making capping efficiency a key indicator of mRNA quality.^[^ ^11^ ^]^ Capping efficiency analysis is required by the United States Pharmacopeia (USP) for mRNA therapeutics.^[^ ^12^ ^]^ Conventionally, capping efficiency is measured by advanced techniques such as LC‐MS, often requiring customized nucleic acid probes.^[^ ^13^ ^]^ However, the complexity of LC‐MS instrumentation and the need for skilled technicians make it impractical for decentralized or on‐site manufacturing. As a result, researchers have been exploring alternative methods, but these either suffer from low sensitivity or still depend on specialized probes.^[^ ^14^ ^]^

**Figure 3:**
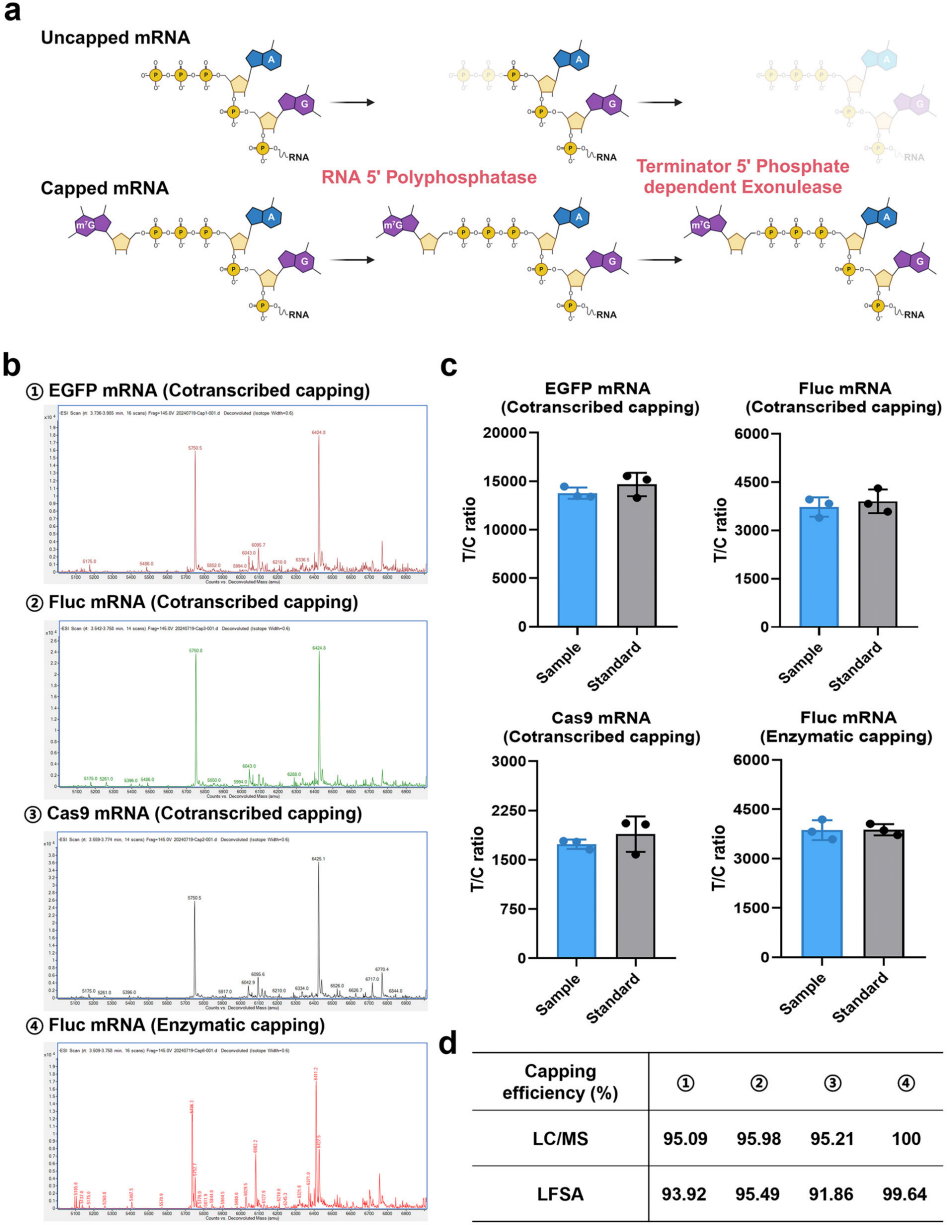
5′ capping efficiency analysis of mRNA. a) Schematic illustration of the enzymatic treatment of mRNA samples. b) Mass spectrometry analysis of cleaved oligonucleotides derived from GAG(3′OMe)‐capped EGFP, Fluc, and Cas9 mRNAs, as well as Fluc mRNA capped using vaccinia capping enzyme. c) Capping efficiency analysis of mRNAs with known sequences using LFSA (*n* = 3), including EGFP, Fluc, and Cas9 (co‐transcriptionally capped) and Fluc (post‐transcriptionally capped). d) Comparative analysis of mRNA capping efficiency measured by LC‐MS and LFSA. Panel a was created with BioRender.com.

To address these limitations, we employed LFSA combined with enzymatic treatment to evaluate mRNA capping efficiency (**Figure** **3**a). Accurate assessment with LFSA relies on a reference signal and a straightforward calculation. Capping efficiency is defined as the ratio of capped mRNA (intact or otherwise) to the total mRNA population, including both capped and uncapped molecules. However, the LFSA alone generates a signal (S_sample_) representing the ratio of fully intact capped mRNA to total mRNA population (Methods). Therefore, to obtain an accurate reference, the mRNA sample was treated with enzymes that selectively degrade uncapped mRNA. After treatment, the LFSA generates a second signal (S_standard_) representing the ratio of fully intact capped mRNA to all capped mRNA. Finally, we can determine the capping efficiency by calculating the signal ratio of S_sample_ to S_standard_ (Experimental Section).

To validate this analytical approach, we conducted several experiments using mRNA samples with varying lengths, sequences, and capping chemistries (Figure 3b,c; Figure S6, Supporting Information). We first tested four mRNAs with known sequences using standard LC‐MS: EGFP, Fluc, and Cas9 mRNAs with co‐transcriptional capping, and Fluc mRNA capped using vaccinia capping enzyme (Methods). The test used 100 pmol of mRNA and combined RNase H cleavage with mRNA enrichment via magnetic beads (Table S1, Supporting Information). After analysis, three possible cleavage sequences (16, 17, and 18 nt) were identified, and their theoretical cleavage ions were predicted based on the RNase H cleavage site (Table S2, Supporting Information). After calculation, the total capping efficiencies of the three co‐transcriptionally capped mRNAs exceeded 95%, while Fluc mRNA capped by vaccinia capping enzyme reached 100% (Figure 3b and Tables S3–S6, Supporting Information).

The LFSA was simple, requiring only 100 ng of mRNA samples and enzyme‐treated standards, with the ratio of their signals representing the capping efficiency (Figure 3c; Figure S6a, Supporting Information). The above results demonstrated a good concordance between these two methods (Figure 3d). Subsequently, we employed the aforementioned methodology to test the Fluc mRNA of unknown sequences from four manufacturers (Figures S5 and S6b, Supporting Information). The results were consistent with the capping efficiency of >95% provided by the manufacturers. Notably, LC‐MS cannot analyze unknown mRNA sequences, whereas LFSA can, highlighting a key advantage of our test strips.

### 2.4 Monitoring the Integrity of mRNA by LFSA

The integrity of mRNA is a critical determinant of the efficacy of mRNA‐based vaccines and therapeutics, as any fragmentation or degradation can severely impair its ability to produce the desired protein within cells. According to the USP, CE is the current standard for mRNA integrity analysis.^[^ ^12^ ^]^ However, CE is a complex and time‐consuming technique that requires skilled operators. Its precision is within a 10% coefficient of variation (CV), while its accuracy is within 20% of the nominal concentration, which limits its broader applications.^[^ ^15^ ^]^ Furthermore, other methods like gel electrophoresis provide only semi‐quantitative data and lack the sensitivity required for high‐precision assessments.^[^ ^16^ ^]^

**Figure 4:**
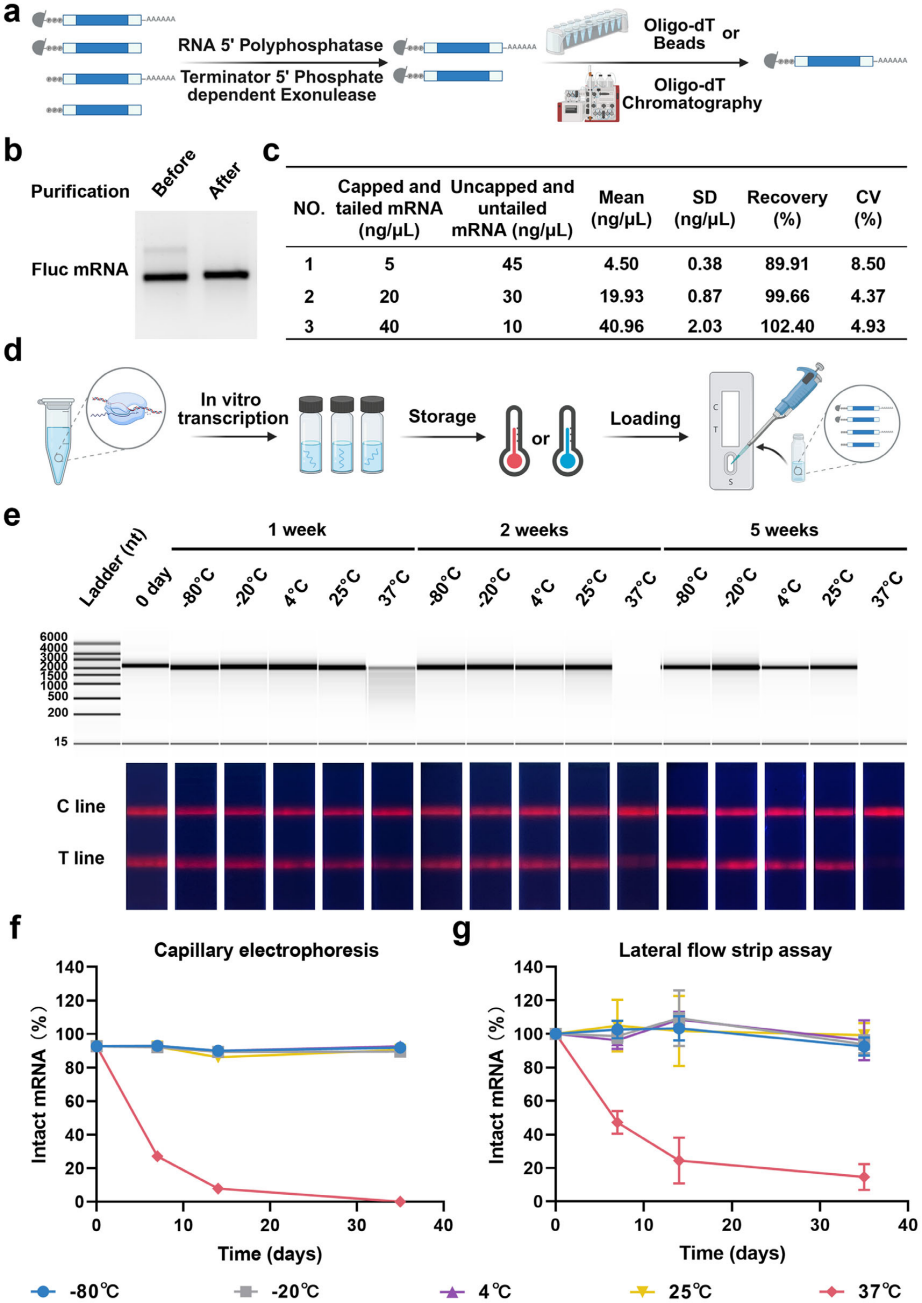
Validation of mRNA integrity analysis. a) Workflow for the purification of structurally intact mRNA. b) Analysis of Fluc mRNA before and after purification using 1% agarose gel electrophoresis. c) Recovery efficiencies and coefficients of variation for detecting capped and tailed Fluc mRNA using LFSA (*n* = 5). d) Schematic illustration of the LFSA for monitoring degradation of Fluc mRNA solution (1 µg µL^−1^) after storage at −80, −20, 4, 25, or 37°C for various durations. e) Capillary electrophoresis analysis of Fluc mRNA solution (1 µg µL^−1^) stored under different conditions, along with photographs of test strips under UV illumination. f) Quantification of mRNA integrity (% intact mRNA) based on CE analysis. g) Relative mRNA integrity (% intact mRNA) measured by the LFSA (*n* = 3). Panels a and d were created with BioRender.com.

To overcome these limitations, we investigated whether our LFSA could serve as a more accurate, real‐time, and user‐friendly approach for evaluating mRNA integrity in decentralized manufacturing settings. A prerequisite for this application was the establishment of a reference point representing intact mRNA. We therefore employed an enzymatic treatment coupled with a purification method to selectively purify mRNA samples with complete integrity and capped structures (**Figure** **4**a,b). This ≈100% integrity sample served as the reference against which other mRNA samples could be evaluated (Figure 4b).

We then conducted a spiked recovery experiment. Specifically, we used intact mRNA at low, medium, and high concentrations, and doped uncapped and untailed mRNA as standards for spiked experiments (Figure 4c). This experiment demonstrated that the LFSA had an accurate quantification ability for intact mRNA. In addition, the LFSA showed a CV below 10% for low‐concentration mRNA quantification and below 5% for medium and high concentrations. These results demonstrated that the LFSA has superior accuracy and precision in mRNA integrity analysis compared to conventional methods like CE. Another experiment showed that CE was less effective than LFSA in distinguishing intact from fragmented or degraded mRNA (Figure 1c; Figure S7, Supporting Information), indicating that LFSA provides higher reliability in detecting intact mRNA.

We also applied LFSA to monitor mRNA integrity during long‐term storage, examining degradation in stock solutions at temperatures from −80 to 37°C over 0–5 weeks (Figure 4d). Fluorescence signal values were recorded at each time point and temperature. For benchmarking, we compared mRNA degradation trends measured by LFSA and CE using reference Fluc mRNA. We established the sensitivity by mapping the detection range for Fluc mRNA, with the limit of detection (LOD) found to be 0.54 ng µL^−1^ for CE and slightly higher at 1.94 ng µL^−1^ for LFSA (Figure S8, Supporting Information). Despite the higher LOD, the LFSA showed comparable sensitivity and a wider detection range than CE, proving its practicality for real‐time mRNA integrity monitoring.

Next, we directly compared mRNA integrity measured by LFSA and CE under various storage conditions (Figure 4e). The mRNA integrity was expressed as a percentage of the initial signal at day 0, which was set to 100% integrity. Over 5 weeks at 37 °C, we observed a gradual decline in mRNA integrity, whereas minimal changes occurred at lower temperatures. Degradation trends detected by LFSA closely matched those obtained by CE (Figure 4f,g; Figure S9, Supporting Information), demonstrating LFSA's reliability for real‐time monitoring of mRNA integrity across different storage conditions.

To validate LFSA against conventional methods, we conducted accelerated thermal degradation tests using EGFP, Fluc, and Cas9 mRNAs at 70 and 80 °C (Figure S10, Supporting Information). Agarose gel electrophoresis provided semi‐quantitative results via ImageJ but was limited by RNase‐related degradation and low sensitivity, particularly for longer mRNAs such as Cas9. HPLC provided high resolution andprecise quantification but required toxic solvents, was time‐consuming, and depended on expensive instrumentation. In contrast, LFSA provided rapid, robust, and portable detection, making it more suitable for decentralized applications.

### 2.5 LFSA Assesses Encapsulation Efficiency and mRNA Concentration in LNPs

According to the USP, the encapsulation efficiency of mRNA in LNPs is a critical parameter for evaluating mRNA vaccines, as it directly influences the delivery and protection of mRNA molecules.^[^ ^17^ ^]^ The Ribogreen fluorescence assay is a currently recommended method for determining encapsulation efficiency.^[^ ^7^ ^]^ We demonstrate that LFSA provides a reliable alternative method for evaluating encapsulation efficiency and mRNA concentration in LNP formulations.

**Figure 5:**
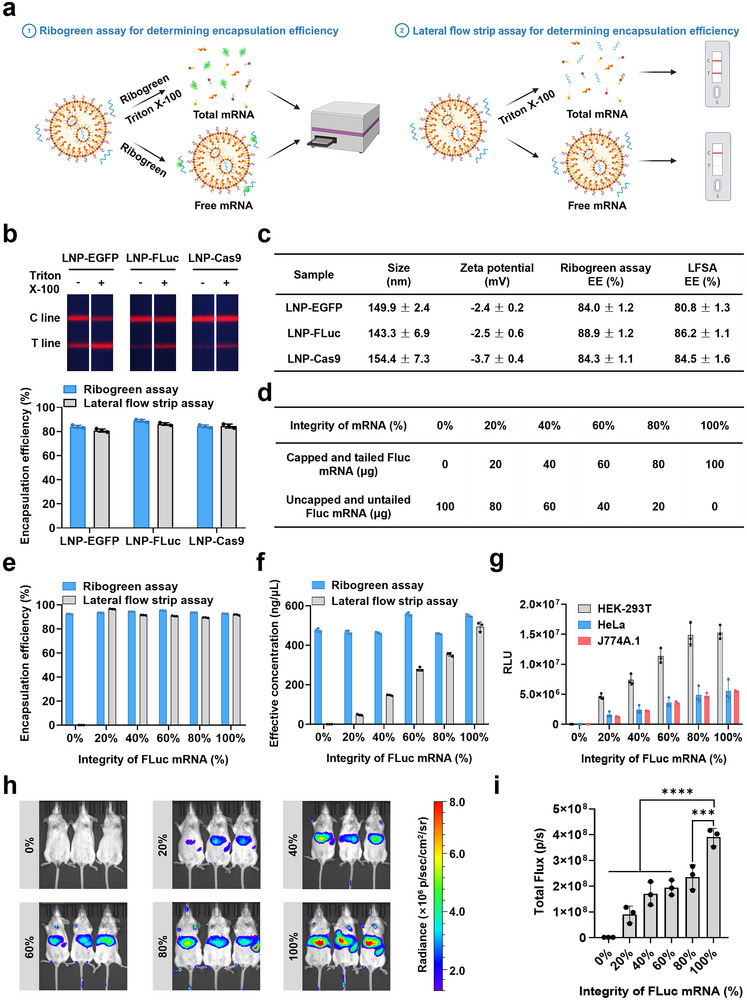
Comparative evaluation of LFSA and Ribogreen assay for determining encapsulation efficiency and mRNA concentration in LNP‐mRNA formulations. a) Schematic illustration of the LFSA and Ribogreen assay used to determine the encapsulation efficiency of LNP‐mRNA. b) Photographs of test strips from three LNP‐mRNA samples treated with or without Triton X‐100 and comparison of encapsulation efficiency using the LFSA and Ribogreen assay (*n* = 3). c) Characterization of three LNP‐mRNA samples, including particle size, zeta potential, and encapsulation efficiency (EE) (*n* = 3). d) Summary table of 100 µg total mRNA composed of varying proportions of intact and fragmented Fluc mRNA. e) Comparative analysis of the encapsulation efficiency of mRNA in LNPs with different doping ratios using the LFSA and Ribogreen assay (*n* = 3). f) Comparative analysis of the effective concentration of mRNA in LNPs with different doping ratios using the LFSA and Ribogreen assay (*n* = 3). g) Validation of cellular expression from LNPs containing Fluc mRNA at various doping ratios in HEK293T, HeLa, and J774A.1 cells (*n* = 3). h) Bioluminescence imaging performed 6 h after intravenous injection of 5 µg LNPs containing Fluc mRNA at different doping ratios into BALB/c mice (*n* = 3 per group). i) Quantification of bioluminescence signals from (h). Data are presented as mean ± SEM, *n* = 3. *P*‐values were calculated using one‐way ANOVA with Dunnett's test, ***p < 0.001, ****p < 0.0001. Panel a was created with BioRender.com.

To highlight the advantages of LFSA, we performed a comparative analysis with the conventional Ribogreen assay (**Figure** **5**a). In the Ribogreen assay, the fluorescence of the LNP‐mRNA solution was measured both with and without the addition of Triton X‐100, which disrupts the LNP structure, allowing for the quantification of encapsulated and free mRNA. Standard curves of Ribogreen fluorescence versus mRNA concentration were used to determine total and free mRNA, enabling calculation of encapsulation efficiency (Figure S11a–c, Supporting Information).

For LFSA, Triton X‐100 also enhanced fluorescence signals. Therefore, we developed standard curves for LFSA both in the presence and absence of 0.2% (v/v) Triton X‐100 to accurately calculate encapsulation efficiency (Figures S11d–f and S12, Supporting Information). The encapsulation efficiencies of LNP‐EGFP, Fluc, and Cas9 mRNA were then determined using LFSA and compared to the Ribogreen assay results. Our experiments showed that LFSA measurements closely matched the results from the Ribogreen assay, confirming the LFSA's reliability for determining mRNA encapsulation efficiency in LNPs (Figure 5b,c).

Additionally, we investigated whether the LFSA buffer could cause LNPs destabilization, which might interfere with the encapsulation efficiency measurement (Figure S13 and Table S7, Supporting Information). Through a series of experiments, we found that the buffer caused a slight reduction in particle size, as indicated by dynamic light scattering (DLS) measurements. However, this reduction was due to the disruption of the hydration membrane by sodium chloride rather than demulsification of the LNPs (Figure S13c, Supporting Information). Interestingly, the addition of Triton X‐100 caused a broadening of the particle size distribution (Figure S13d, Supporting Information), indicating demulsification, which was consistent with our findings from the Ribogreen assay.

Next, we tested the LFSA's ability to differentiate between intact and incomplete mRNA molecules encapsulated in LNPs. Samples were prepared by mixing intact mRNA with uncapped and untailed mRNA in proportions ranging from 0% to 100% intact mRNA (Figure 5d). Encapsulation efficiencies and mRNA concentrations were measured using both LFSA and the Ribogreen assay. While the Ribogreen assay showed high encapsulation efficiency (over 90%) regardless of the proportion of intact mRNA, LFSA demonstrated 0% encapsulation efficiency for samples containing only uncapped and untailed mRNA (Figure 5e). This result confirmed that LFSA is highly specific for detecting intact mRNA, providing a more accurate reflection of effective mRNA concentration in LNPs (Figure 5f).

We further validated LFSA's advantages by testing Fluc mRNA expression in three different cell types, observing that mRNA expression levels were directly proportional to the amount of intact mRNA, as measured by LFSA (Figure 5g). This positive correlation was consistent with in vivo results, where mRNA expression in mice after intravenous injection closely matched LFSA‐based predictions (Figure 5h,i). We attempted to assess LNP‐mRNA encapsulation using agarose gel electrophoresis. mRNA was visible only after Triton X‐100 treatment, while untreated samples remained in the well with no clear free mRNA band, suggesting limited sensitivity of this method for detecting the encapsulation efficiency (Figure S14, Supporting Information). These results highlight LFSA's ability to objectively and accurately assess LNP‐mRNA functionality, establishing it as a valuable tool for mRNA vaccine development and quality control.

### 2.6 On‐Site QC of mRNA by LFSA

LNP‐mRNA vaccines are highly sensitive to temperature fluctuations.^[^ ^18^ ^]^ Improper storage conditions can lead to mRNA degradation, thereby diminishing vaccine potency. An immediate, on‐site QC system is crucial for real‐time monitoring of mRNA vaccine quality. Such a system enables the early detection of deviations from optimal storage conditions, ensuring that only effective doses are administered to patients.

**Figure 6:**
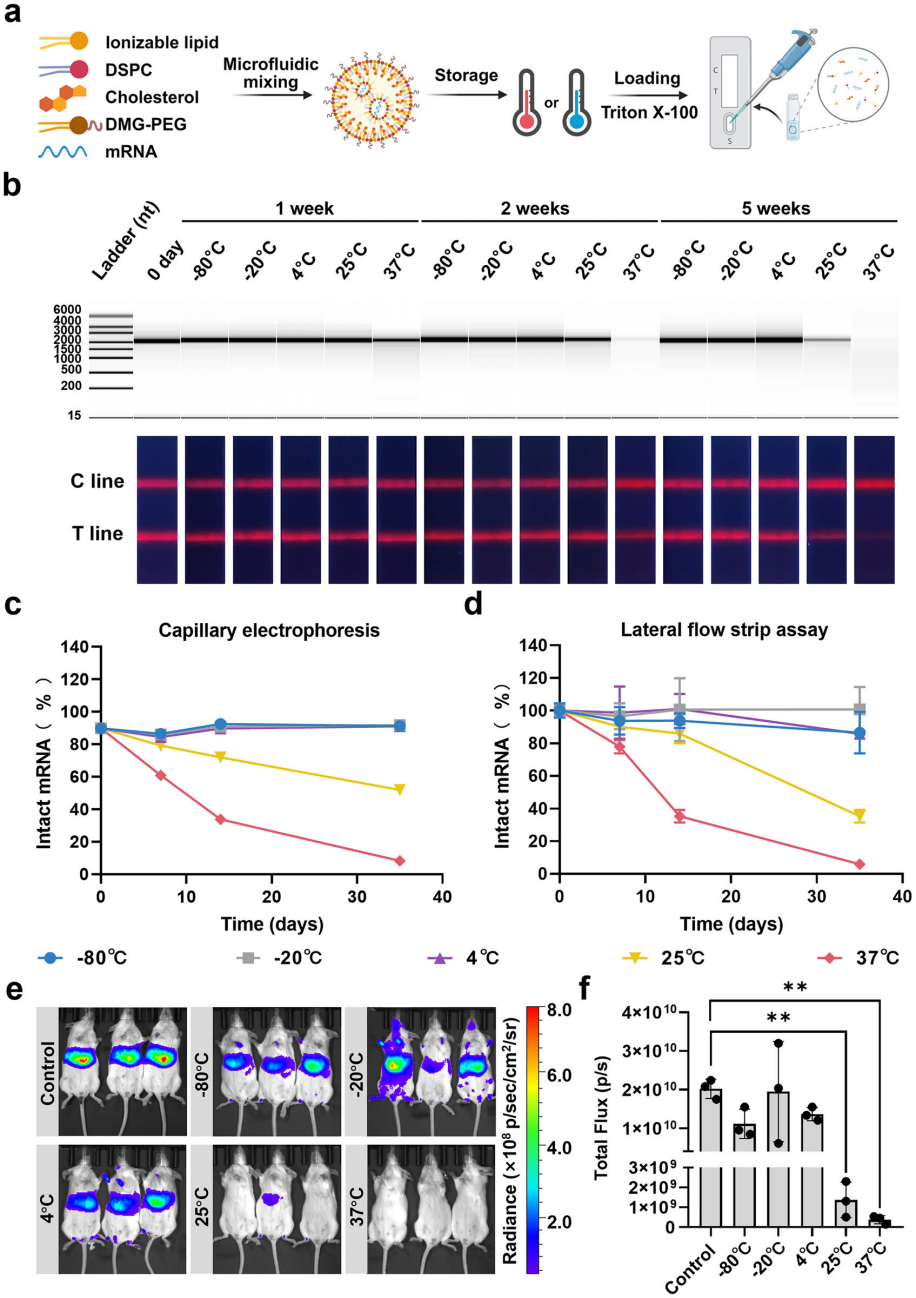
On‐site QC of LNP‐mRNA by LFSA. a) Schematic illustration of LFSA for monitoring degradation of Fluc mRNA (100 ng µL^−1^) encapsulated in LNPs after storage at −80, −20, 4, 25, or 37°C for various durations. b) Capillary electrophoresis analysis of Fluc mRNA extracted from LNPs after storage under the indicated conditions, along with photographs of corresponding strips under UV illumination. c) Integrity of mRNA in LNPs (% intact mRNA) determined by capillary electrophoresis. d) Integrity of mRNA in LNPs (% intact mRNA) determined by LFSA and quantified using an immunofluorescence analyzer (*n* = 3). e) Bioluminescence imaging performed 6 h after intravenous injection of 5 µg LNP‐Fluc mRNA, stored under the specified conditions, into BALB/c mice (*n *= 3 per group). f) Quantification of bioluminescence signals from (e). Data are presented as mean ± SEM, *n* = 3. *P*‐values were calculated using one‐way ANOVA with Dunnett's test, **p < 0.01. Panel a was created with BioRender.com.

Previous findings have indicated that mRNA degrades faster than lipid compositions in LNP formulations.^[^ ^19^ ^]^ Therefore, monitoring mRNA integrity serves as a reliable indicator of the overall condition of mRNA vaccines. To demonstrate LFSA's feasibility for on‐site QC, we assessed mRNA integrity in LNPs using a standard SM‐102 formulation (Figure S15, Supporting Information). To ensure that LNPs can be stored at low temperatures, we used 8% sucrose as the cryoprotectant. The LNP‐mRNA samples were diluted and tested under the same conditions as the mRNA stock, with the addition of Triton X‐100 to facilitate demulsification before testing (**Figure** **6**a).

These results revealed detectable mRNA degradation in LNPs stored at elevated temperatures, specifically, after 5 weeks at 25°C and 2 weeks at 37 °C (Figure 6b–d; Figure S16, Supporting Information). While LNPs provided protection at −80, −20, and 4 °C, higher temperatures led to mRNA degradation. We found that when naked mRNA was stored at 25 °C for 5 weeks, the mRNA did not undergo significant degradation, yet when LNP‐mRNA was stored at 25 °C, the mRNA was slightly degraded for the second week. This might be because naked mRNA was stored directly in RNase‐free water and RNase‐free centrifuge tubes after synthesis, whereas LNP‐mRNA was degraded in LNPs due to the multiple complex steps required for synthesis and dialysis, and the whole process might introduce RNase into the LNP solution. A comparative analysis using CE demonstrated that LFSA captured similar degradation trends, reinforcing its utility for monitoring mRNA stability in LNPs. In addition to assessing mRNA integrity, we analyzed the physicochemical properties of LNPs. Measurements included DLS, PDI, and zeta potential. These data indicated increased particle sizes and PDI values, along with more negative zeta potentials, suggesting potential mRNA leakage and degradation (Figure S17, Supporting Information). These physicochemical changes confirmed the degradation observed in LFSA and CE analyses. Finally, we intravenously injected LNP‐Fluc mRNA, stored under different conditions, into BALB/c mice, demonstrating a direct correlation between mRNA integrity and bioluminescence intensity (Figure 6e,f). This validates LFSA as an effective tool for assessing LNP‐mRNA integrity.

To further assess LFSA performance versus conventional methods, we conducted accelerated degradation experiments by incubating LNP‐encapsulated EGFP or Fluc mRNA at 70 and 80 °C for 1 h. The integrity results obtained from LFSA were highly consistent with those from agarose gel electrophoresis, confirming its analytical reliability (Figure S18a–c, Supporting Information). Notably, EGFP fluorescence intensity in HEK293T cells decreased more substantially than the overall LNP transfection efficiency (Figure S18d–g, Supporting Information), suggesting that mRNA degradation or functional loss occurs before the decline in LNP delivery capability. Moreover, we observed that LNP‐mRNA formulations tended to aggregate under thermal or prolonged storage conditions, which correlated with strong retention in agarose gels and resistance to Triton X‐100 disruption. Although mRNA might remain intact, such aggregates rendered it functionally inactive, as reflected by the loss of LFSA signals. Finally, in vivo bioluminescence imaging revealed markedly reduced signals after injection of thermally degraded LNP‐Fluc mRNA (Figure S18h–i, Supporting Information), further supporting LFSA as a reliable and practical indicator of mRNA vaccine functionality.

## 3 Discussion

We have developed a multifunctional LFSA designed to evaluate key quality attributes of mRNA, including capping efficiency, integrity, encapsulation efficiency, and effective concentration. The LFSA stands out for its simplicity and practicality, as it does not require elaborate preparation, sophisticated machinery, or professional technical expertise.

The LFSA is designed to detect specific mRNA features, such as the 5' cap and poly(A) tail, offering significant advantages over traditional techniques. By combining LFSA with enzymatic digestion, it accurately measures mRNA capping efficiency regardless of sequence, modifications, or capping methods. The LFSA also enables real‐time monitoring of mRNA integrity and degradation, providing quick, reliable data in just 15 min, which is especially valuable for preparing high‐quality mRNA for cell‐based or animal experiments. Unlike the Ribogreen assay, which cannot distinguish between intact and degraded mRNA, the LFSA accurately assesses both encapsulation efficiency and effective mRNA concentration in LNPs. This ensures that mRNA vaccines are correctly formulated and maintain their functional integrity, which is critical for their therapeutic efficacy.

Throughout this study, we rigorously compared the performance of the LFSA against current industry‐standard techniques, including LC‐MS, capillary electrophoresis, and the Ribogreen assay. We summarized the comparison of key parameters in Tables S8–S10 (Supporting Information). To our knowledge, this is the first lateral flow strip assay capable of simultaneously assessing three mRNA quality attributes without sequence‐specific probes or complex sample preparation. LFSA is rapid (≤100 ng sample, minimal preparation), low‐cost (portable devices, 5 min instruction; Table S11, Supporting Information), and maintains high resolution (5%), accurate quantification (±10%), and strong precision (CV<10%). These features make it ideally suited for decentralized mRNA vaccine manufacturing and real‐time quality control, including in low‐ and middle‐income settings.

Although the LFSA demonstrates high specificity and universality for assessing mRNA quality attributes, several limitations remain. Chemical modifications can improve mRNA stability and translation, but may reduce assay sensitivity by affecting probe binding. Different encapsulation materials, such as polymers, virus‐like particles, and novel lipid nanoparticles, may interfere with mRNA release and probe access. Moreover, stable long‐chain mRNA reference standards for integrity and quantitative analysis are lacking. To address these challenges, future studies should standardize sample pre‐treatment by optimizing thermal denaturation or adding auxiliary proteins (e.g., helicases) to facilitate mRNA unfolding and release. In addition, strategies such as engineered RNA polymerases should be used to synthesize stable long‐chain reference mRNAs, improving the reliability and reproducibility of quantitative analysis.

In conclusion, the development of a comprehensive mRNA QC system represents a major advance in mRNA therapeutics. It offers a practical, efficient, and reliable tool for ensuring the quality of mRNA vaccines, enabling researchers and manufacturers alike to produce and verify mRNA that meets industry‐grade standards. As the demand for mRNA vaccines continues to grow, the LFSA will play a pivotal role in supporting the rapid, safe, and effective deployment of these vital technologies.

## 4 Experimental Section

### Labelling Antibodies with FNPs

Antibody‐conjugated FNPs were synthesized via the amidization coupling reaction.^[^ ^20^ ^]^ In brief, 100 µL of 1% (wt/v) carboxylated FNPs was diluted with 800 µL of 50 mM MES buffer (pH 6.0). Subsequently, 45 µL of NHS (100 mg mL) and 15 µL of EDC (100 mg mL^−1^) were added to the mixture, which was then incubated on a rotary mixer at room temperature for 30 min. Subsequently, the surface‐activated nanoparticles were centrifuged at 18 753 ×g for 10 min and re‐suspended in 1 mL of 50 mM MES buffer (pH 6.0). Anti‐m^7^G rabbit monoclonal antibody was added to the activated nanoparticles at a concentration of 50 µg per 500 µL, and the reaction proceeded for 1 h at room temperature. Subsequently, 15 µL of the blocking solution, containing 15% (wt/v) BSA and 0.1% (v/v) ProClin, was added to the mixture, and the blocking reaction proceeded for 1 h. Finally, the mixture was centrifuged at 18,753×g at 4 °C for 10 min. The FNPs‐antibody conjugates were resuspended in 50 µL of 10 mM PBS buffer (5% (wt/v) sucrose, 1% (wt/v) BSA, 0.5% (v/v) Tween‐20, 0.1% (v/v) ProClin 300, pH 7.4).

### Preparation Procedures of Lateral Flow Strips

1) Treatment of test strips: The sample pad was soaked in 10 mM PBS buffer (0.5% (wt/v) BSA, 0.05% (v/v) Tween‐20, pH 7.4) for 5 min, then dried at 25°C and with a humidity of less than 20% for one day. The conjugation pad was soaked in 10 mM PBS buffer (5% (wt/v) sucrose, 1% (wt/v) BSA, and 0.5% (v/v) Tween‐20, pH 7.4) for 5 min, then dried at 25°C and with a humidity of less than 20% for one day. The FNPs‐antibody conjugates were applied to the conjugation pad at a rate of 2 µL cm^−1^ using an XYZ 3D film spraying instrument. The samples were then dried at 25 °C for 5 h. A solution of 25 µM streptavidin and 25 µM biotin‐modified poly(dT_25_) was prepared in 50 mM HEPES buffer (100 mM KCl, pH 7.4). The DNA mixture was applied to the T line on the NC membrane at a rate of 1 µL cm^−1^. Meanwhile, 0.1 mg mL^−1^ of anti‐rabbit IgG antibody was sprayed onto the C line on the NC membrane at a rate of 1 µL cm^−1^. The NC membrane was then dried at 25°C for 5 h. 2) Assembly of test strips: The components, namely the absorption pad, conjugation pad, sample pad, and NC membrane, were assembled on the polyvinyl chloride (PVC) adhesive pad in a specific sequence, with each part overlapping by 2 mm. The NC membrane was positioned at the bottom. The assembled card was then cut into 3 mm‐wide strips using a Strip cutter‐CTS300. These strips were subsequently stored in a desiccator at room temperature.

### Preparation of In Vitro Transcription Template

The plasmids encoded the T7 promoter, followed by a variety of genes, including CD40, NanoLuc, mCherry, mRuby2, EGFP, CD40L, OVA, Fluc, Cas13a, Spike, and Cas9. Additionally, they contained 5' UTR α‐globin and 3' UTR α‐globin. Polymerase chain reaction (PCR) was conducted using the 2×Phanta Flash Master Mix in conjunction with 0.4 µM of each forward and reverse primer (Table S12, Supporting Information) to amplify the plasmid insert. During the amplification process, a polydT tail comprising 120 thymidines was incorporated into the plasmid insert.

### mRNA In Vitro Transcription and Purification

The synthesis of mRNA was conducted via IVT, as previously described.^[^ ^21^ ^]^ Co‐transcriptional capping reactions were conducted in 20 µL reactions comprising 1×transcription buffer (20 mM MgCl_2_, 2.5 mM TCEP, and 2 mM spermidine in 40 mM Tris‐HCl (pH 8.0)), 500 ng IVT template, and 2.5 U µL^−1^ T7 RNA polymerase, 0.01 U µL^−1^ inorganic pyrophosphatas, 5 mM each NTP, and 4 mM cap structure analog were utilized in the synthesis. The transcription reactions were incubated at 37°C for 2 h and subsequently treated with a final concentration of 0.04 U µL DNase I for 30 min at 37 °C. Enzymatic capping reactions were conducted in a solution of 1×capping Buffer, 10 µg denatured RNA, 0.5 U µL^−1^ vaccinia capping enzyme, 2.5 U µL^−1^ 2'‐O‐methyltransferase, 0.5 mM GTP, and 0.2 mM SAM. The reactions were incubated at 37 °C for 1 h. The synthesized mRNA was purified using magnetic beads. The transcription reactions yielded two distinct capping methods (co‐transcriptional capping and enzymatic capping) and two types of cap structure analogs, m7G(5')ppp(5')(2'OMeA)pG and m7(3'OMeG)(5')ppp(5') (2'OMeA)pG. The following modifications were made to the mRNA: U, Ψ, m^1^Ψ, m^5^C/Ψ, and mo^5^U. Additionally, eleven types of sequence‐encoded mRNAs were generated, with their description, nucleotide length, and molar mass listed in Table S13 (Supporting Information).

### General Lipid Nanoparticles Synthesis

The lipid nanoparticles were prepared by mixing one volume of the following lipid composition (SM‐102, DSPC, cholesterol, and DMG‐PEG2000) dissolved in ethanol at mole ratios of 50:10:38.5:1.5, with three volumes of mRNA (amine‐to‐phosphate ratio of 6) suspended in 10 mM citrate buffer (pH 3.0). The lipids in ethanol solution and the mRNA prepared in citrate buffer were introduced into microfluidic mixers at a flow rate ratio of 1:3, respectively, with a combined final flow rate of 12 mL min^−1^. The flow rate for the ethanol was 3 mL min^−1^, while that for the aqueous buffer was 9 mL min^−1^. The solution was then subjected to dialysis overnight against 1×PBS in a Pur‐A‐Lyzer Midi 3500 Dialysis tube at 4 °C. The LNPs were concentrated through Amicon Ultra Centrifugal Filters, dispersed in a solution of 20 mM Tris‐HCl buffer (8% (wt/v) sucrose, pH 7.4), and stored at 4°C before use.

### Capping Efficiency Measurement by LC‐MS

92 µL of the RNA sample (100 pmol) was mixed with 4 µL of cleavage probe (50 µM). In a thermocycler, the samples were heated to 95 °C for 2 min, subsequently cooled from 70 to 16°C at 0.1°C s^−1^. After the annealing cycle, 12 µL of 10×RNase H reaction buffer and 16 µL of RNase H (5 U µL^−1^) were added to the tube containing 92 µL of products. The samples were incubated for 20 min at 25 °C. Finally, the cleaved fragments were purified by magnetic beads, followed by analysis using the Agilent 6545XT AdvanceBio LC/Q‐TOF system.

### Explore the Effect of mRNA Structure on LFSA Results

To test the strips, 100 ng of mRNA (CD40, NanoLuc, mCherry, mRuby2, EGFP, CD40L, OVA, Fluc, Cas13a, Spike, or Cas9 mRNAs) was mixed with 50 µL of loading buffer (0.2% NP40, 3% NaCl, and 2% formamide in 10 mM Tris‐HCl (pH 7.4)) and applied to the sample well of the strip. Following a 15‐min wait, all fluorescence signals were measured using an immunofluorescence analyzer with an excitation wavelength of 365 nm and an emission wavelength of 618 nm. The T/C ratio was determined by calculating the signal intensity at the T line and dividing it by that at the C line. This ratio was then multiplied by a factor of 2000. Standard curves were constructed for EGFP, Fluc, and Cas9 mRNA using different amounts (0, 5, 10, 15, 25, 50, 100 ng) of mRNA relative to strips' signals (T/C ratio). To further investigate the influence of mRNA structure on LFSA performance, standard curves were constructed to evaluate chemical modifications, capping chemistry, and sequence composition of mRNAs.

### LFSA for Testing the Capping Efficiency of mRNA

To test the capping efficiency by LFSA, it was first necessary to remove uncapped mRNA. 2 µg of mRNA were incubated with 10 units of RNA 5' polyphosphatase in a final volume of 10 µL containing 1×RNA 5' polyphosphatase buffer for 45 min at 37 °C. Subsequently, mRNA samples were treated with 0.5 units of the Terminator 5' phosphate‐dependent exonuclease in a final volume of 20 µL, which contained 1×Terminator 5' phosphate‐dependent exonuclease buffer A, for 45 min at 30 °C. To serve as a control, an identical buffer system and procedure were employed, except for the enzymatic treatment. mRNAs were purified using magnetic beads, and the concentrations were determined with a NanoDrop One spectrophotometer. The enzyme‐treated mRNA was used as the standard, and the untreated mRNA as the sample. By calculating the signal ratio of two mRNA samples in the test strip, we can determine the capping efficiency. The capping efficiency was estimated using the following formula:

(1) $$Loading\;amount\;\left(ng\right)=m_{a}+m_{b}+m_{c}+m_{d}=m_{A}+m_{B}$$

(2) $$\frac{m_{a}}{m_{a}+m_{b}}=\frac{m_{A}}{m_{A}+m_{B}}\;↔\;\frac{m_{a}}{m_{A}}=\frac{m_{a}+m_{b}}{m_{A}+m_{B}}$$

(3) $$\begin{array}{ccc} Capping\;efficiency\;\left(\%\right) & = & \frac{m_{a}+m_{b}}{m_{a}+m_{b}+m_{c}+m_{d}}*100\%\\ & = & \frac{m_{a}+m_{b}}{m_{A}+m_{B}}*100\%=\frac{m_{a}}{m_{A}}*100\%\\ & = & \frac{S_{sample}}{S_{standard}}*100\% \end{array}$$

Of which, *a*, *b*, *c*, and *d* are the four components in the mRNA sample before enzyme treatment, respectively. *A* and *B* are the two components in the mRNA standard after enzyme treatment, respectively. S_sample_ is the signal value of mRNA sample. S_standard_ is the signal value of mRNA standard. m_a_ is the amount of capped and tailed mRNA in the sample, m_b_ is the amount of capped and untailed mRNA in the sample, m_c_ is the amount of uncapped and tailed mRNA in the sample, m_d_ is the amount of uncapped and untailed mRNA in the sample, m_A_ is the amount of capped and tailed mRNA in the standard, m_B_ is the amount of capped and untailed mRNA in the standard. It needs to be emphasized that the loading amount of the sample and the standard must be the same.

### Preparation of ≈100% Intact mRNA

To obtain ≈100% intact mRNA, mRNA samples were first treated with RNA 5' polyphosphatase and Terminator 5' phosphate‐dependent exonuclease to remove all uncapped RNA. The obtained samples were then purified three times by magnetic beads containing poly(dT_25_) to obtain complete mRNA. If the amount of mRNA sample is large enough, magnetic beads can be replaced by FPLC for mRNA purification.

### Measurement of mRNA Integrity using Capillary Electrophoresis Analysis

The purity of mRNA was measured on Agilent Fragment Analyzer 5200 with RNA kit (15 NT). 2 µL of mRNA samples (20 ng µL^−1^) were mixed with 22 µL of diluent, followed by heating at 70 °C for 2 min, then chilled on ice for 5 min. To assess the sensitivity of capillary electrophoresis for mRNA detection, a standard curve was constructed for different concentrations of Fluc mRNA relative to relative fluorescence units (RFUs). The formula of LOD is as follows: LOD = 3σ/S, where σ is the standard deviation of the intercept, and S is the slope of the calibration curve. For mRNA testing in LNPs, LNP‐mRNA samples were first treated with 0.2% (v/v) Triton X‐100 to disrupt particles, and subsequent steps were unchanged. A 6000‐nt RNA ladder from the kit was used as the marker to estimate the size of mRNA. The pre‐set RNA method was used for separation. ProSize 2.0 software was used to process the raw data.

### Measurement of mRNA Integrity Using Ion Pair Reverse‐Phase High‐Performance Liquid Chromatography (IP‐RP‐HPLC)

*m*RNA samples were analyzed by HPLC (Agilent 1260, USA) using a 250 × 4 mm reverse‐phase LC column with a particle size of 5 µm and a pore size of 4000 Å (Agilent Technologies: PLRP‐S 4000Å). The chromatographic analysis was performed using the following conditions: Buffer A: 0.1 m hexyl ammonium acetate (HAA); Buffer B: 0.1 M HAA in 50% acetonitrile. The gradient started at 30% buffer B and remained for 10 min, followed by a linear extension to 100% buffer B over 10 min, then maintained at 100% buffer B over 10 min. The system flow rate was set as 0.2 mL min^−1^, and the column temperature was 60 °C. mRNA was detected by UV absorbance at 260 nm.

### LFSA for Assessing the Integrity of mRNA Stock Solution

To assess the sensitivity of LFSA for mRNA detection, a standard curve was constructed for different concentrations of Fluc mRNA relative to signals (T/C ratio). The formula of LOD is the same as that used by CE for mRNA detection. To display the test strip's capacity to monitor alterations in mRNA integrity, 1 µg µL^−1^ of Fluc mRNA was stored at various temperatures (ranging from −80 to 37 °C) for a designated period. Subsequently, 1 µL of mRNA was diluted tenfold, mixed with 49 µL of sampling buffer, and applied to the test strip. After 15 min, the value was read by the machine, with day 0 values set as 100% integrity. This approach enabled the comparative assessment of mRNA integrity across different temperatures and time points.

### LFSA for On‐Site QC of mRNA

To assess the mRNA stability in LNPs under diverse conditions, LNPs containing Fluc mRNA at a concentration of 100 ng µL^−1^ were stored at varying temperatures (ranging from −80 to 37 °C) for designated durations. Subsequently, an equal volume of 0.4% Triton X‐100 was added to disrupt the emulsion. 2 µL of the resulting solution was mixed with 48 µL of loading buffer and applied to the test strip. The relative integrity of mRNA in LNP formulations across different temperatures and time points was assessed through comparative analysis.

### Measurement of mRNA Encapsulation Efficiency using the Ribogreen Assay

As previously described, the mRNA encapsulation efficiency and concentration were determined using the Quant‐iT RiboGreen Assay. The quantification of mRNA in LNPs was conducted using a standard curve generated from a dilution series of the corresponding mRNA stock. Both the standards and the samples were diluted with 1×phosphate‐buffered saline (PBS). Fluorescence was quantified using the SpectraMAX M5 fluorescence microplate reader, with an excitation wavelength of 485 nm and an emission wavelength of 530 nm. The standard curve was calculated by linear regression analysis of the fluorescence intensity plotted against the concentration of the standard. The mRNA encapsulation of LNP samples was determined by comparing the signal of RiboGreen in the absence and presence of a detergent (0.2% (v/v) Triton X‐100). In the absence of a detergent, the signal is derived exclusively from unencapsulated mRNA. Conversely, in the presence of a detergent, LNPs are disrupted, resulting in a signal that represents the total mRNA (both encapsulated and non‐encapsulated). The encapsulation efficiency is calculated using the following equation:

(4) $$\begin{array}{ccc} & & Encapsulation\;efficiency\;\left(\%\right)\\ & & =\frac{\left[Disrupted\;LNP\;mRNA\;Conc.\right]-\left[Undisrupted\;LNP\;mRNA\;Conc.\right]}{Disrupted\;LNP\;mRNA\;Conc.}\\ & & \;\times 100\% \end{array}$$

### LFSA for Testing the Encapsulation Efficiency and Effective Concentration of mRNA in LNPs

The standard curves for mRNA were initially constructed based on its mass and T/C ratio. Given the potential for Triton X‐100 to influence the signal, two separate standard curves were generated, one with Triton X‐100 and one without, for mRNA quantification. The encapsulation efficiency was calculated using the following equation:

(5) $$\begin{array}{ccc} & & Encapsulation\;efficiency\;\left(\%\right)\\ & & =\frac{\left[Disrupted\;LNP\;mRNA\;Conc.\right]-\left[Undisrupted\;LNP\;mRNA\;Conc.\right]}{Disrupted\;LNP\;mRNA\;Conc.}\\ & & \;\times 100\% \end{array}$$

To facilitate comparison with the Ribogreen assay, we encapsulated varying percentages (0%, 20%, 40%, 60%, 80%, and 100%) of capped and tailed Fluc mRNA using SM‐102 LNPs. The encapsulation efficiency was evaluated using the previously described method, and the effective encapsulation dose was determined using standard curves established by the aforementioned method. The significance of the effective encapsulated dose of mRNA in LNP was verified using Fluc mRNA expression experiments in three cell types and animal experiments.

### Fluc mRNA Expression Assay

In vitro expression of Fluc mRNA was conducted in 48‐well culture plates, with 200 µL/well of 0.5×10⁵‐1.0×10⁵ cells seeded and treated with each LNP containing Fluc mRNA (200 ng mRNA/well). Fluc expression was quantified using the TransDetect Single‐Fluc (Firefly) Reporter Assay Kit after 24 h. The luminescence was measured using the SpectraMAX M5 fluorescence microplate reader.

### Administration of LNP‐mRNA to Mice

Female BALB/c mice (≈8 weeks old) were administered intravenous injections of SM‐102 LNPs encapsulating Fluc mRNA (*n* = 3 per group). In brief, 5 µg of LNP‐mRNA in PBS were intravenously injected. Subsequently, after 6 h, mice were administered 150 mg kg^−1^ of D‐Luciferin intraperitoneally. Following the administration of D‐luciferin for 10 min, the mice were anesthetized with isoflurane and transferred to the imaging platform. Bioluminescence imaging was conducted using the IVIS Spectrum imaging system. The photon flux (photons/second) within the region of interest, from which the bioluminescence signal originated, was quantified using the Living IMAGE Software provided by Caliper.

### Cells and Animals

The cell lines HEK293T (Human Embryonic Kidney 293 Cells; ATCC), HeLa (Human Cervical Cancer Cells; ATCC), and J774A.1 (Mouse Mononuclear Macrophage Cells; ATCC) were maintained in high‐glucose DMEM, supplemented with 100 U mL^−1^ penicillin and 100 µg mL^−1^ streptomycin, and 10% fetal bovine serum at 37°C in 5% CO_2_. Female BALB/c mice (≈8 weeks old) were procured from Vital River Co. (Beijing, China) and maintained under pathogen‐free conditions. All animal experiments were conducted by a protocol (NCNST21‐202410‐0116) approved by the Animal Research Committee of the National Center for Nanoscience and Technology, Chinese Academy of Sciences (Beijing, China).

### Statistics Analysis

The data were subjected to statistical analysis using Prism 8.0 software (GraphPad, San Diego, CA). All data were presented as means ± standard deviation (SD) of at least three independent experiments. Statistical comparisons were performed using one‐way ANOVA with Dunnett's test. **p < 0.01; ***p < 0.001; ****p < 0.0001.

## Conflict of Interest

The authors declare no conflict of interest.

## Author Contributions

D.W., J.Z., and Y.C. designed the experiments. D.L., Z.Z., G.J., A.H., Q. C., and J.Z. conducted the experiments and performed the data analysis. D.W., Y.Y., and Y.C. were responsible for research supervision, coordination, and strategy. D.W. and Y.C. drafted the manuscript. D.W., Y.Y., and Y.C. reviewed and edited the manuscript. All authors reviewed and approved the final version of the manuscript.

## Supporting information



Supporting Information

## Data Availability

The data that support the findings of this study are available from the corresponding author upon reasonable request.

## References

[1] A. Kumar , J. Blum , T. T. Le , N. Havelange , D. Magini , I. K. Yoon , Nat. Rev. Drug Discovery 2022, 21, 333. 35149859 10.1038/d41573-022-00035-z

[2] S. Qin , X. Tang , Y. Chen , K. Chen , N. Fan , W. Xiao , Q. Zheng , G. Li , Y. Teng , M. Wu , X. Song , Signal Transduct. Target. Ther. 2022, 7, 166. 35597779 10.1038/s41392-022-01007-w PMC9123296

[3] L. Schoenmaker , D. Witzigmann , J. A. Kulkarni , R. Verbeke , G. Kersten , W. Jiskoot , D. J. A. Crommelin , Int. J. Pharm. 2021, 601, 120586. 33839230 10.1016/j.ijpharm.2021.120586 PMC8032477

[4] O. J. Wouters , K. C. Shadlen , M. Salcher‐Konrad , A. J. Pollard , H. J. Larson , Y. Teerawattananon , M. Jit , Lancet 2021, 397, 1023. 33587887 10.1016/S0140-6736(21)00306-8 PMC7906643

[5] S. Fahrag , S. A. Peña‐Benavides , L. Thiel , C. Sengoba , K. Karacasulua , N. Ihling , J. E. Sosa‐Hernández , G. Gilleskie , J. M. Woodley , R. Parra‐Saldivar , S. S. Mansouri , K. Roh , Ind. Eng. Chem. Res. 2022, 61, 13191.

[6] C. Hu , Y. Bai , J. Liu , Y. Wang , Q. He , X. Zhang , F. Cheng , M. Xu , Q. Mao , Z. Liang , Expert Rev. Vaccines 2024, 23, 570. 38733272 10.1080/14760584.2024.2354251

[7] L. J. Jones , S. T. Yue , C. Y. Cheung , V. L. Singer , Anal. Biochem. 1998, 265, 368. 9882416 10.1006/abio.1998.2914

[8] a) M. M. Ali , M. Mukherjee , K. Radford , Z. Patel , A. Capretta , P. Nair , J. D. Brennan , Angew. Chem., Int. Ed. 2023, 135, 202307451; 10.1002/anie.202307451 37477970

[9] a) T. Masek , V. Vopalensky , P. Suchomelova , M. Pospisek , Anal. Biochem. 2005, 336, 46; 15582557 10.1016/j.ab.2004.09.010

[10] a) D. M. Mauger , B. J. Cabral , V. Presnyak , S. V. Su , D. W. Reid , B. Goodman , K. Link , N. Khatwani , J. Reynders , M. J. Moore , I. J. McFadyen , Proc. Natl. Acad. Sci. USA 2019, 116, 24075; 31712433 10.1073/pnas.1908052116 PMC6883848

[11] M. Warminski , A. Mamot , A. Depaix , J. Kowalska , J. Jemielity , Acc. Chem. Res. 2023, 56, 2814. 37782471 10.1021/acs.accounts.3c00442 PMC10586375

[12] USP‐NF , https://www.uspnf.com/notices/analytical‐procedures‐mrna‐vaccines‐20240802 (accessed: October 2024).

[13] M. Beverly , A. Dell , P. Parmar , L. Houghton , Anal. Bioanal. Chem. 2016, 408, 5021. 27193635 10.1007/s00216-016-9605-x

[14] a) S. Chiron , P. H. Jais , J. Genet. Genomics 2017, 1, 46;

[15] Agilent DNF‐471 (15nt) https://www.agilent.com/en/product/automated‐electrophoresis/fragment‐analyzer‐systems/fragment‐analyzer‐systems‐rna‐analysis‐kits/fragment‐analyzer‐rna‐kits‐365734 (accessed: April 2024).

[16] a) S. Chiron , P. H. Jais , J. Genet. Genomics 2017, 1, 46;

[17] a) K. N. Kafetzis , N. Papalamprou , E. McNulty , K. X. Thong , Y. Sato , A. Mironov , A. Purohit , P. J. Welsby , H. Harashima , C. Yu‐Wai‐Man , A. D. Tagalakis , Adv. Healthcare Mater. 2023, 12, 2203022; 10.1002/adhm.202203022 PMC11468535 36906918

[18] a) P. Zhao , X. Hou , J. Yan , S. Du , Y. Xue , W. Li , G. Xiang , Y. Dong , Bioact. Mater. 2020, 5, 358; 32206737 10.1016/j.bioactmat.2020.03.001 PMC7078456

[19] a) L. Schoenmaker , D. Witzigmann , J. A. Kulkarni , R. Verbeke , G. Kersten , W. Jiskoot , D. J. A. Crommelin , Int. J. Pharm. 2021, 601, 120586; 33839230 10.1016/j.ijpharm.2021.120586 PMC8032477

[20] T. Ji , X. Xu , X. Wang , Q. Zhou , W. Ding , B. Chen , X. Guo , Y. Hao , G. Chen , Sensor Actuat. B‐chem. 2019, 282, 309.

[21] D. Luo , Z. Wu , D. Wang , J. Zhang , F. Shao , S. Wang , S. Cestellos‐Blanco , D. Xu , Y. Cao , Mol. Ther. Nucl. Acids 2023, 32, 445. 10.1016/j.omtn.2023.04.005 PMC10173069 37181450

