# Supplementary material for: A Comprehensive Lateral Flow Strip Assay for On‐Site mRNA Vaccine Quality Control in Decentralized Manufacturing
Source: Adv Sci (Weinh). 2025 Sep 3;12(43):e02387. doi: 10.1002/advs.202502387 (PMC12631917; doi:10.1002/advs.202502387)
Supplement: Supplementary file 1 — Supporting Information [file ADVS-12-e02387-s001.docx]

Supporting Information

A Comprehensive Lateral Flow Strip Assay for On-Site mRNA Vaccine Quality Control in Decentralized Manufacturing

Dengwang Luo, Ziwei Zhang, Jing Guo, Jieli Zhang, Arong Huang, Qinghao Cao, Jiangnan Zheng, Yingjin Yuan, Daming Wang*, and Yuhong Cao*

**Materials and apparatus**

PCR primers were purchased from Tsingke Biotech (Beijing, China). HEPES (catalog number: H8090), streptavidin (catalog number: S9171), and sucrose (catalog number: S8271), 1×PBS (catalog number: P1020) were purchased from Solarbio (Beijing, China). KCl (catalog number: H-10016318), formamide (catalog number: A57268), and Triton X-100 (catalog number: A110694) were purchased from Sangon (Shanghai, China). RNase-free H_2_O (catalog number: ST876) was purchased from Beyotime (Beijing, China). Tris-HCl solution (pH 7.4) (catalog number: SL3090) and 5 M NaCl (catalog number: SL92310) were purchased from Coolaber (Beijing, China). 10% NP40 (catalog number: R21361) was purchased from Yuanye (Shanghai, China). Ribogreen (catalog number: R11490) and inorganic pyrophosphatase were purchased from Thermo Fisher Scientific. 10 mM citrate buffer (pH 3.0) (catalog number: R00540) was purchased from Leagene (Beijing, China). Ethyl alcohol (catalog number: A60719) was purchased from Innochem (Beijing, China). RNA 5' Polyphosphatase (catalog number: RP8092H) and Terminator 5' Phosohate-dependent Exonulease (catalog number: TER51020) were purchased from LGC (Teddington, England). 2×Phanta Flash Master Mix (catalog number: P510), FastPure Gel DNA Extraction Mini Kit (catalog number: DC301), T7 RNA polymerase (catalog number: 4101PC), DNase I (catalog number: EN401), vaccinia capping enzyme (catalog number: DD4109), 2'-O-Methyltransferase (catalog number: DD4110), magnetic beads (catalog number: N412), and D-Luciferin (catalog number: DD1210) were purchased from Vazyme (Nanjing, China). NTPs were purchased from Synthgene (Nanjing, China). DMEM (catalog number: 11965092), FBS (catalog number: A5670701), and Penicillin-Streptomycin (catalog number: 15070063) were purchased from Sigma-Aldrich (St. Louis, MO, USA). Time-resolved fluorescent nanoparticles (catalog number: MF02) were purchased from Meiniu (Changsha, China). DH5α Chemically Competent Cells (catalog number: TSC-C14) were purchased from Tsingke (Beijing, China). Plasmid Mini Kit (catalog number: D6943-01) was purchased from Omega (Georgia, USA). TransDetect® Single-Luciferase (Firefly) Reporter Assay Kit (catalog number: FR101) was purchased from TransGen (Beijing, China). Anti-7-Methylguanosine rabbit monoclonal antibody (catalog number: ab300741) was purchased from Abcam (Cambridge, UK). Anti-Rabbit IgG antibody (catalog number: W4011) was purchased from Genstars (Nanjing, China). 1-octylnonyl 8-[(2-hydroxyethyl)[6-oxo-6-(undecyloxy)hexyl]amino]-octanoate (SM-102, catalog number: 2089251-47-6) was purchased from Avanti (Birmingham, UK). 1,2-dimyristoyl-rac-glycero-3-methoxypolyethylene glycol-2000 (PEG2000-DMG, catalog number: 160743-62-4) was purchased from GuoBang Pharmaceutical (Hangzhou, China). Cholesterol (Chol, catalog number: 57-88-5) and 1,2-distearoyl-sn-glycero-3-phosphocholine (DSPC, catalog number: 816-94-4) were purchased from Ai Wei Tuo (Shanghai, China). Oligo (dT) 25 Beads were purchased from APExBIO (Shanghai, China). The nitrocellulose membrane (NC) was purchased from Millipore (Massachusetts, USA). The sample pad, conjugate pad, bottom plate, and absorbent pad were purchased from Jinbiao (Shanghai, China)

**Material Characterization**

The fluorescence signals of test strips were measured by an Immunofluorescence Analyzer (Anbio, AF-100S, China). The fluorescence and luminescence were measured by the SpectraMAX M5 fluorescence microplate reader (Molecular Device, United States). The hydrodynamic diameters of particles and zeta potentials were measured by a Zetasizer Nano-ZS instrument (Malvern Panalytical, UK). Bioluminescence imaging was conducted using the IVIS Spectrum imaging system (PerkinElmer, USA). The concentration of nucleic acids was determined by the NanoDrop One spectrophotometer (Thermo Scientific, USA). Lipid nanoparticles were produced by microfluidic mixers (Micro&Nano, Minhang District, Shanghai). The XYZ 3D film spraying instrument and spray gold instrument used for preparing the lateral flow strip were purchased from Shanghai Jinbiao Biotechnology Co., Ltd. (Shanghai, China).

**
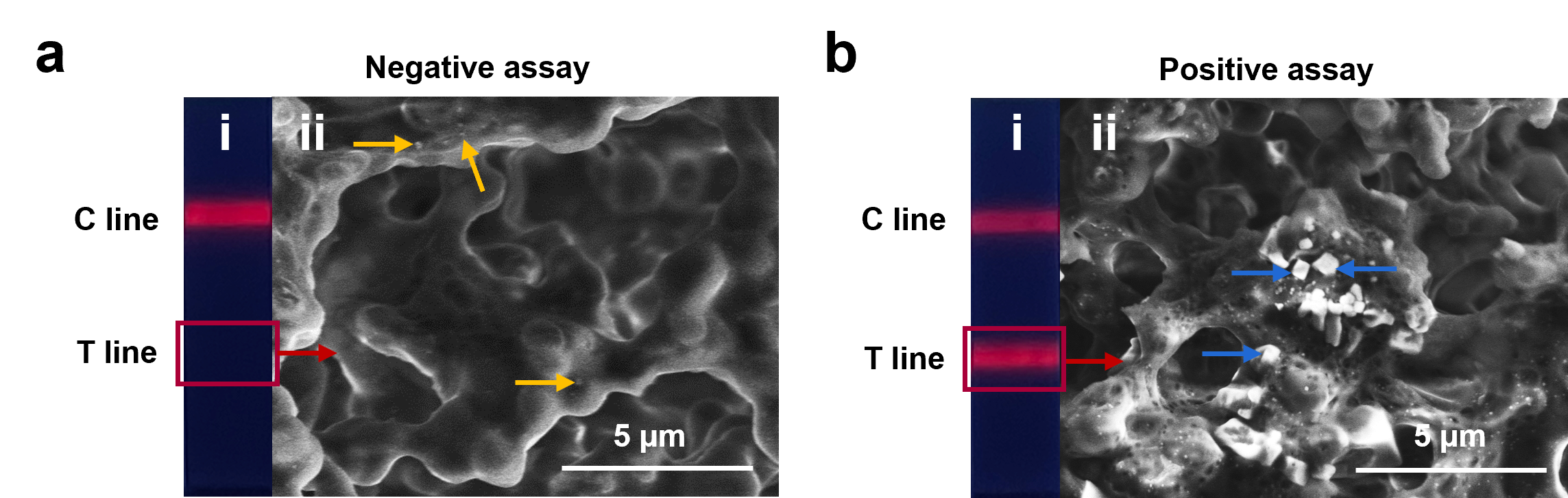
**

**Figure S1.** **Fluorescence (i) and SEM (ii) images of the strip membrane after negative (a) and positive assays (b, 100 ng of Fluc mRNA).** The yellow arrows indicate fluorescence nanoparticles adsorbed on the T-line. The blue arrows indicate mRNA captured on the T-line.

**
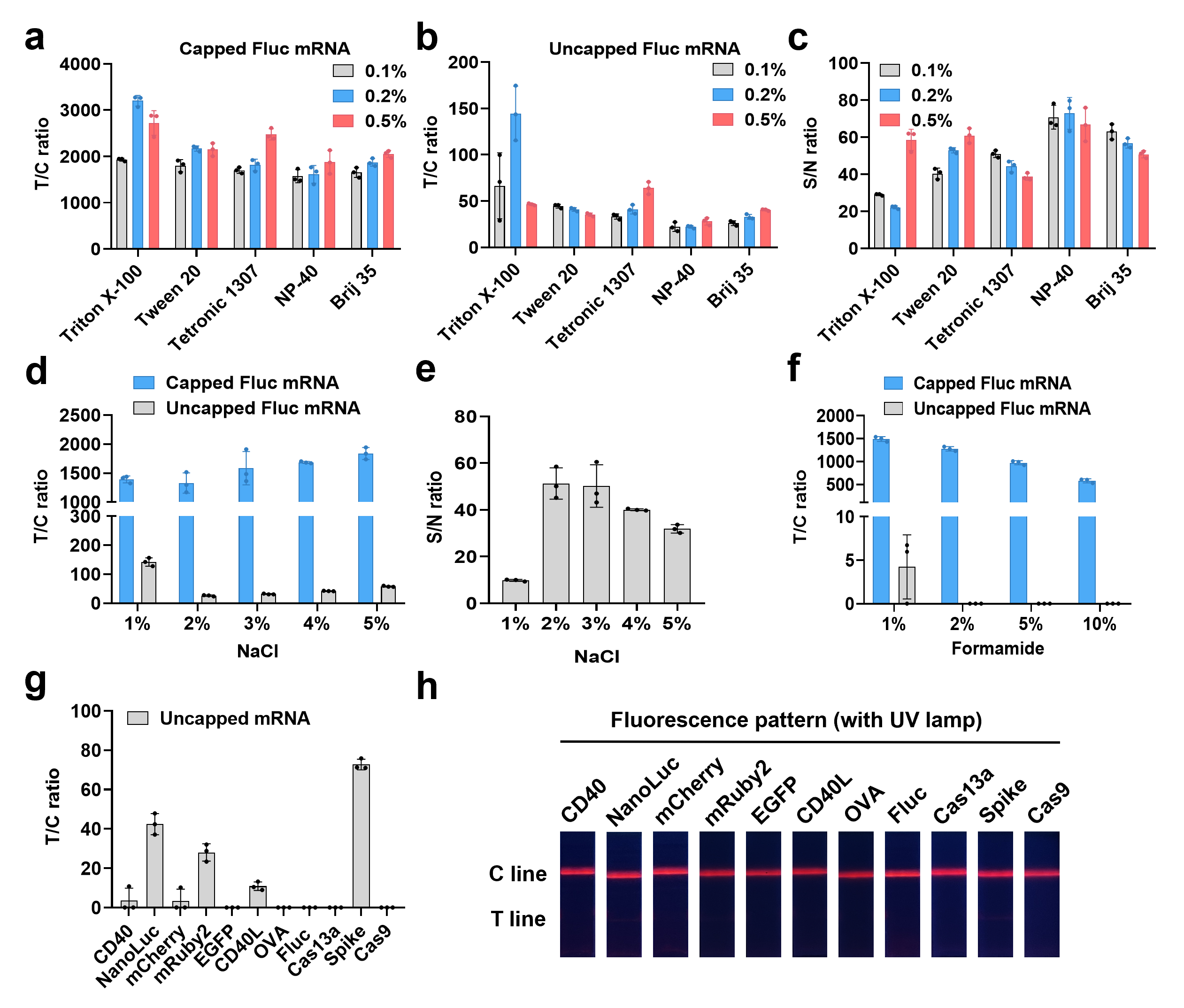
**

**Figure S2. Optimization of the sample buffer.**

**(a)** Specific signals of capped Fluc mRNA diluted in sample buffer (containing various types and ratios of nonionic surfactants) after loading onto the lateral flow strips (n = 3).

**(b)** Nonspecific signals of uncapped Fluc mRNA diluted in sample buffer (containing various types and ratios of nonionic surfactants) after loading onto the lateral flow strips (n = 3).

**(c)** Signal-to-noise (S/N) ratios of Fluc mRNA diluted in sample buffer (containing various types and ratios of nonionic surfactants) after loading onto the lateral flow strips (n = 3).

**(d)** Signals of capped and uncapped Fluc mRNA diluted in sample buffer (containing varying concentrations of sodium chloride) after loading onto the lateral flow strips (n = 3).

**(e)** Signal-to-noise (S/N) ratios of Fluc mRNA diluted in sample buffer (containing varying concentrations of sodium chloride) after loading onto the lateral flow strips (n = 3).

**(f)** Signals of capped and uncapped Fluc mRNA diluted in sample buffer (containing varying concentrations of formamide) after loading onto the lateral flow strips (n = 3).

**(g)** Nonspecific signals of uncapped mRNA (including CD40, NanoLuc, mCherry, mRuby2, EGFP, CD40L, OVA, Fluc, Cas13a, Spike, and Cas9 mRNAs) after loading onto the lateral flow strips (n = 3).

**(h)** Photographs of all test strips taken under a UV lamp.


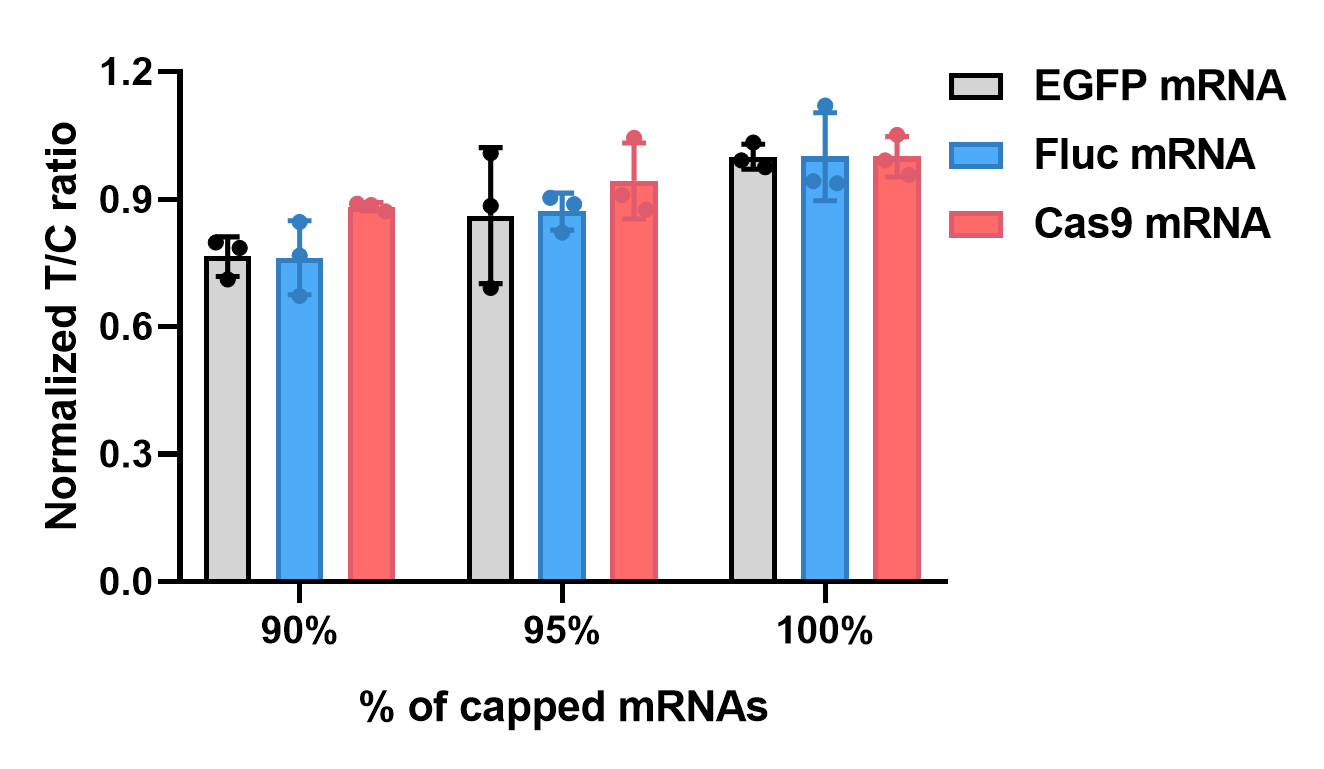


**Figure S3. Investigation of resolution in mRNA quantification.**

Normalized signals of 100 ng of capped mRNAs at different percentages (including EGFP, Fluc, and Cas9 mRNA) after loading onto the strips (n = 3).


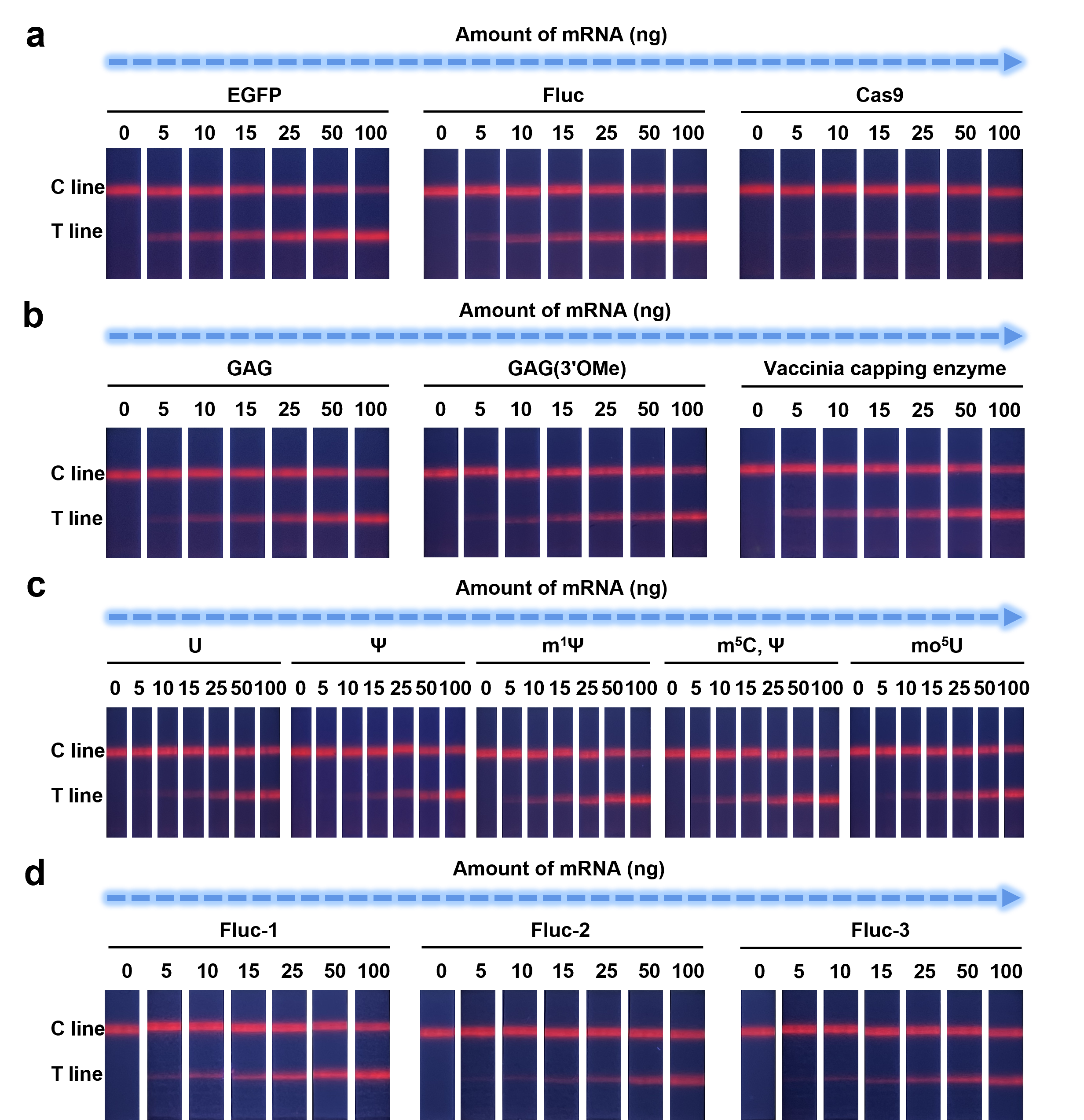


**Figure S4. LFSA results for various capped mRNAs with different modifications and sequences.**

**(a)** Photographs of LFSA results for different percentages of capped mRNAs (including EGFP, Fluc, and Cas9 mRNAs) under a UV lamp.

**(b)** Photographs of LFSA results for different percentages of capped mRNAs (including Fluc mRNAs co-transcriptionally capped with two types of cap analogues and post-transcriptionally capped with vaccinia capping enzyme) under a UV lamp.

**(c)** Photographs of LFSA results for different percentages of capped mRNAs (including Fluc mRNAs modified with U, Ψ, m^1^Ψ, m^5^C/Ψ, and mo^5^U) under a UV lamp.

**(d)** Photographs of LFSA results for different percentages of capped mRNAs (including three different sequences of Fluc mRNAs from Biosyn Biotechnology Co., Ltd.) under a UV lamp.


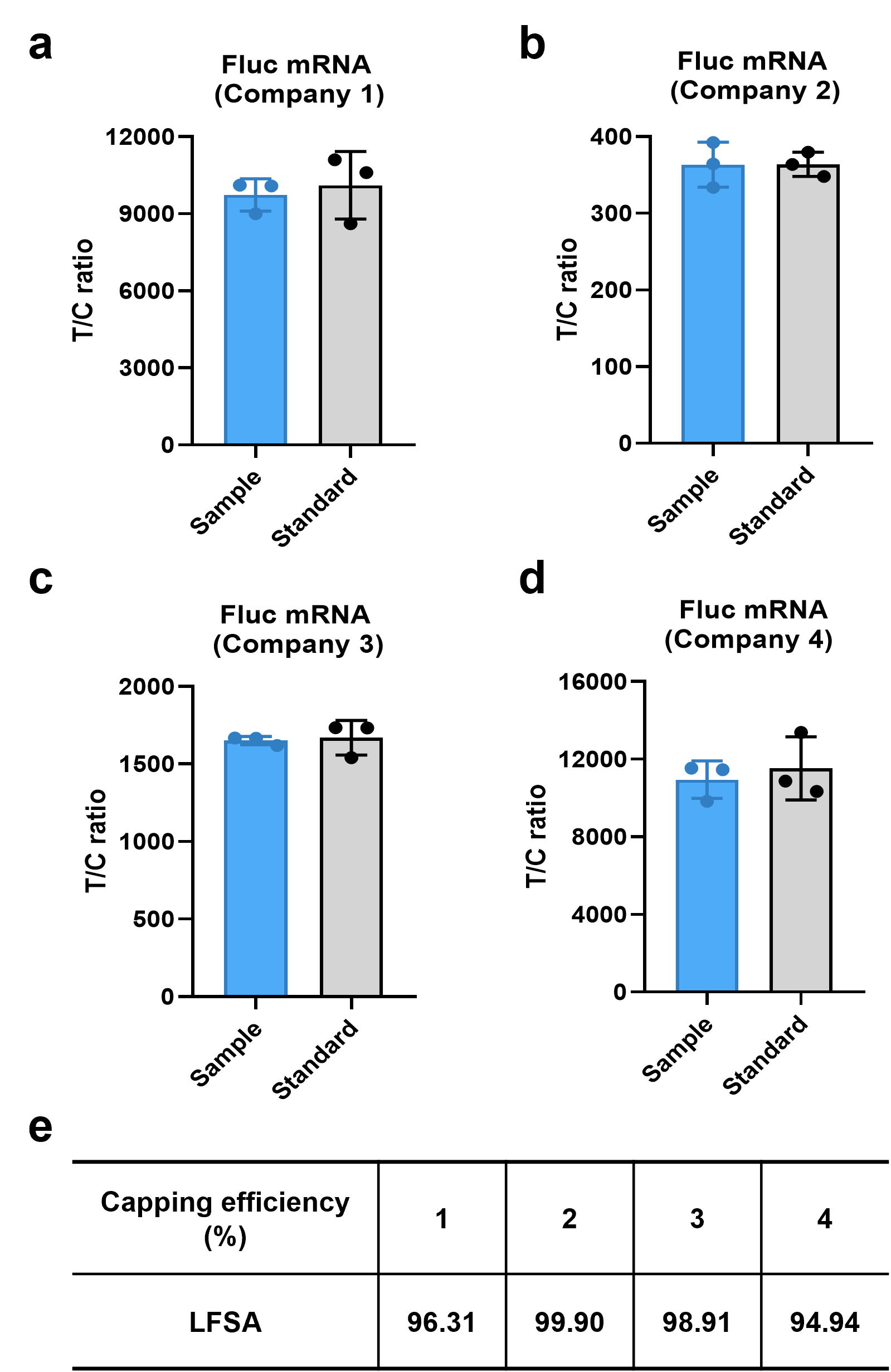


**Figure S5. Capping efficiency analysis of mRNA samples.**

**(a-d)** Capping efficiency analysis of mRNAs with unknown sequences using LFSA (n = 3), including Fluc mRNAs from four different companies.

**(e)** Table of statistical results for mRNA capping efficiency.


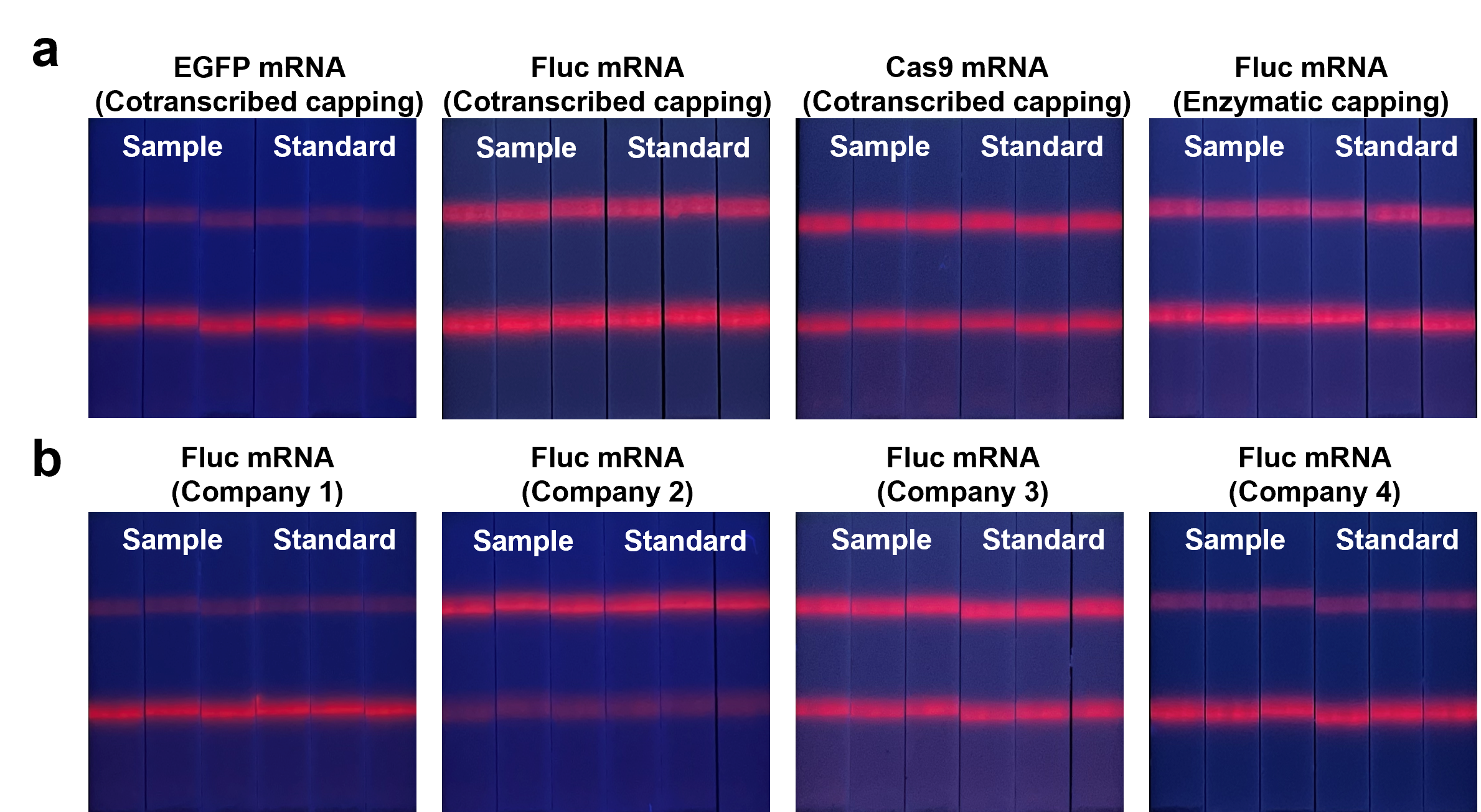


**Figure S6.** **Photographs of test strips assessing capping efficiency of 100 ng mRNA samples under a UV lamp.**

**(a)** Testing mRNA capping efficiency for known sequences (EGFP, Fluc, Cas9 co-transcriptionally capped, and Fluc post-transcriptionally capped) (n = 3).

**(b)** Testing mRNA capping efficiency for unknown sequences, including Fluc mRNAs from four companies (n = 3).


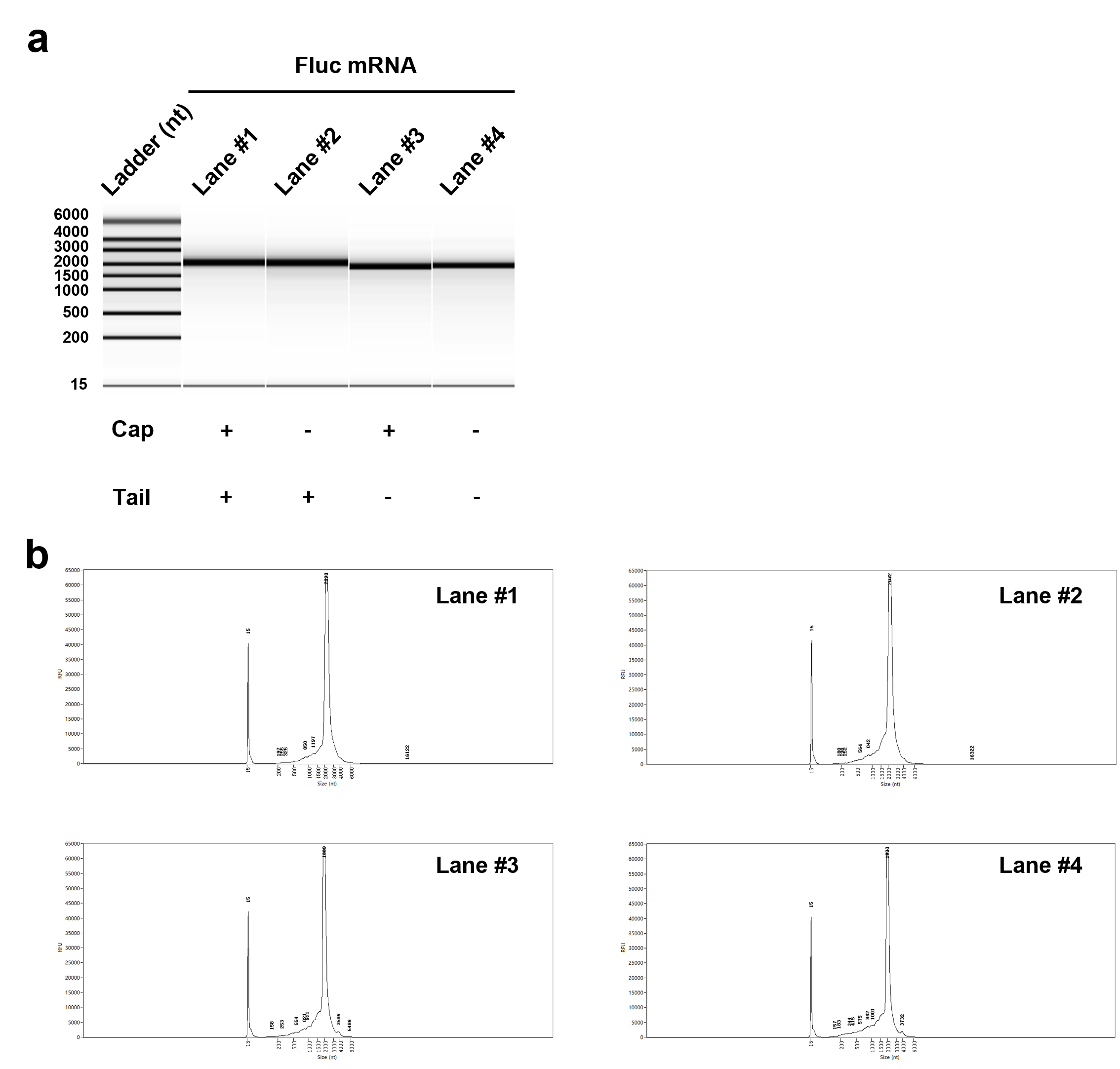


**Figure S7. Capillary electrophoresis analysis of different forms of Fluc mRNA.**

**(a)** Capillary electrophoresis of different forms of Fluc mRNA.

**(b)** Electropherograms of different forms of Fluc mRNA.


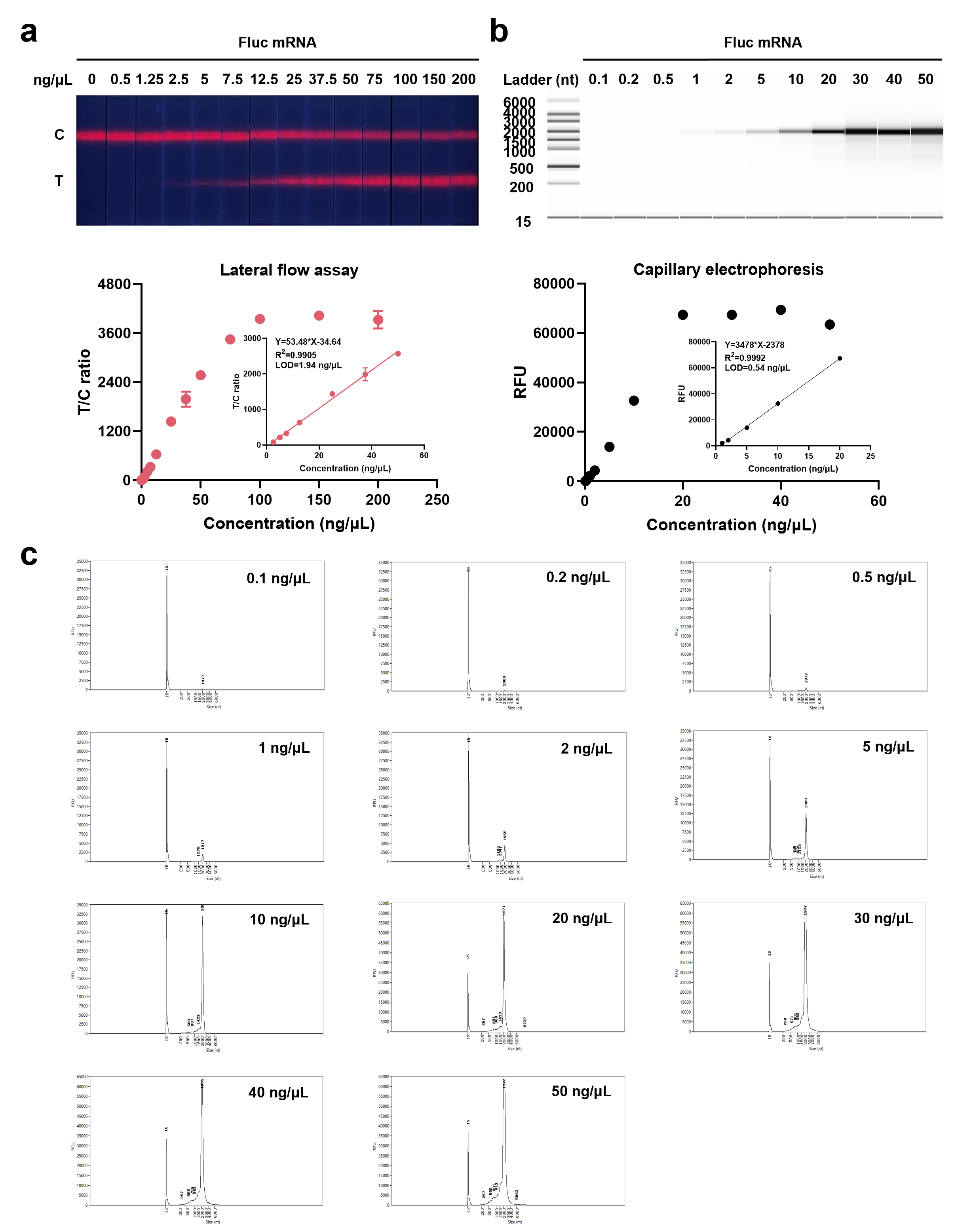


**Figure S8. Comparison of LFSA and capillary electrophoresis for the quantitative analysis of Fluc mRNA.**

**(a)** Photographs of LFSA results for detecting different concentrations of Fluc mRNA. Scatter plot of the fluorescence signal from LFSA as a function of Fluc mRNA concentration. Inset: The linear relationship between fluorescence signal and Fluc mRNA concentration (n = 3).

**(b)** Total electropherograms for different concentrations of Fluc mRNA. Scatter plot of RFU from capillary electrophoresis (CE) analysis as a function of Fluc mRNA concentration. Inset: The linear relationship between RFU and Fluc mRNA concentration.

**(c)** Electropherograms for each concentration of Fluc mRNA.


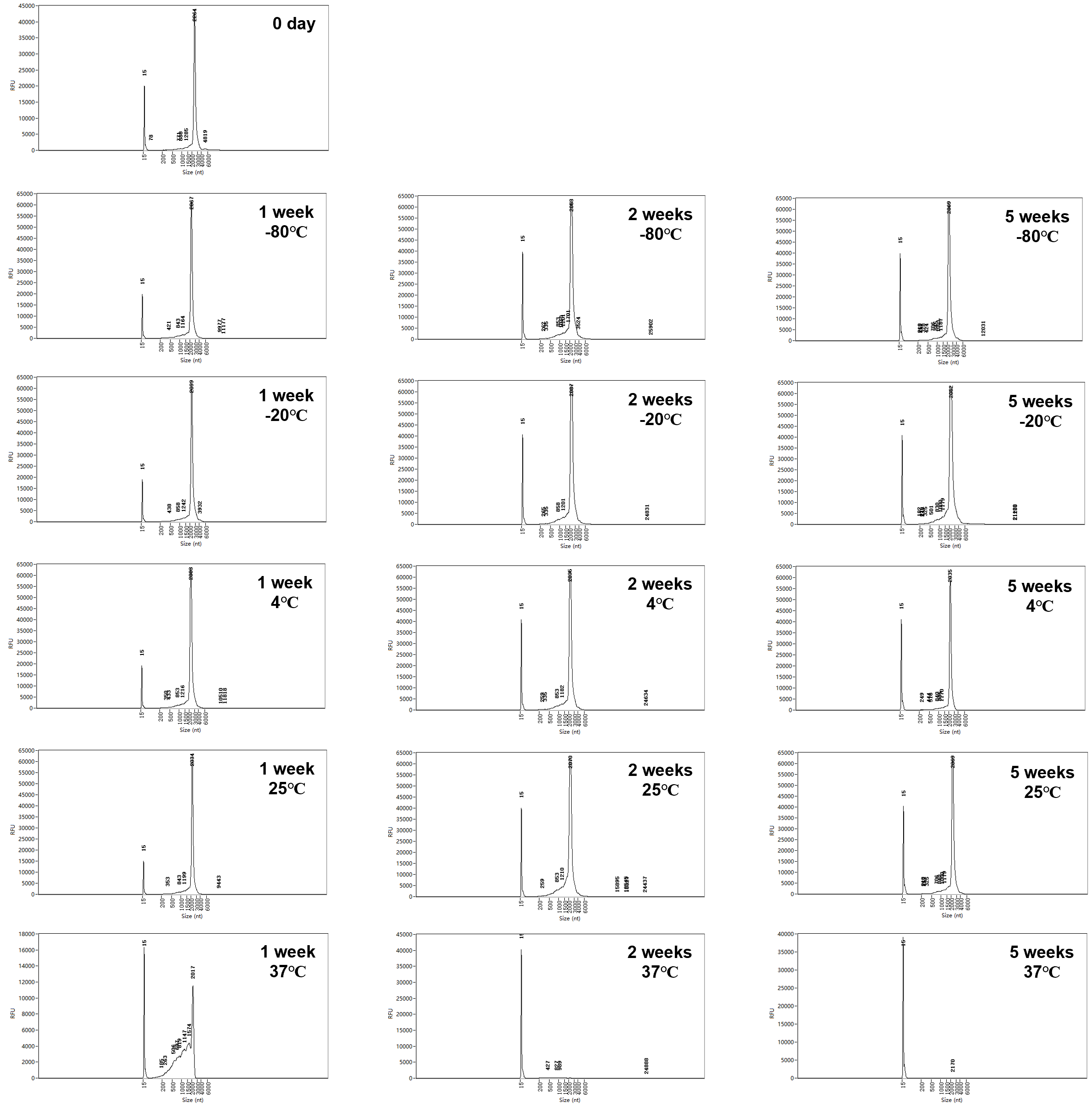


**Figure S9. Electropherograms of Fluc mRNAs after storage at -80°C, -20°C, 4°C, 25°C, or 37°C for various durations.**

**
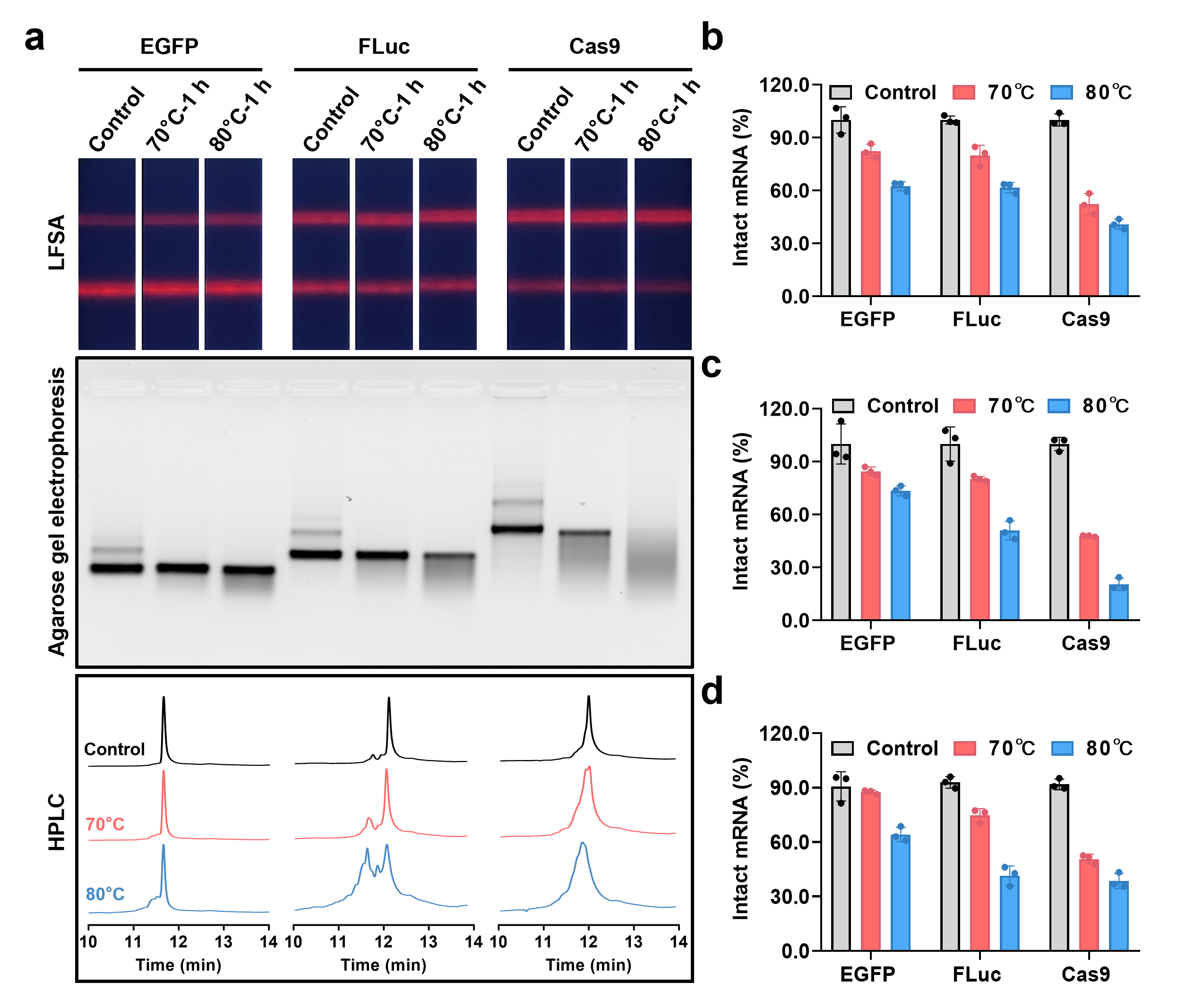
**

**Figure S10. Evaluation of mRNA degradation under elevated temperatures using LFSA, agarose gel electrophoresis, and high-performance liquid chromatography (HPLC).**

**(a)** Degradation analysis of EGFP, Fluc, and Cas9 mRNA (100 ng/μL) under initial conditions and after thermal incubation at 70°C or 80°C for 1 h using LFSA, 1% agarose gel electrophoresis, and HPLC.

**(b)** Quantification of mRNA integrity based on LFSA using an immunofluorescence analyzer (n = 3).

**(c)** Quantification of mRNA integrity from gel electrophoresis images analyzed by ImageJ (n = 3).

**(d)** Quantification of mRNA integrity based on HPLC chromatograms (n = 3).

**
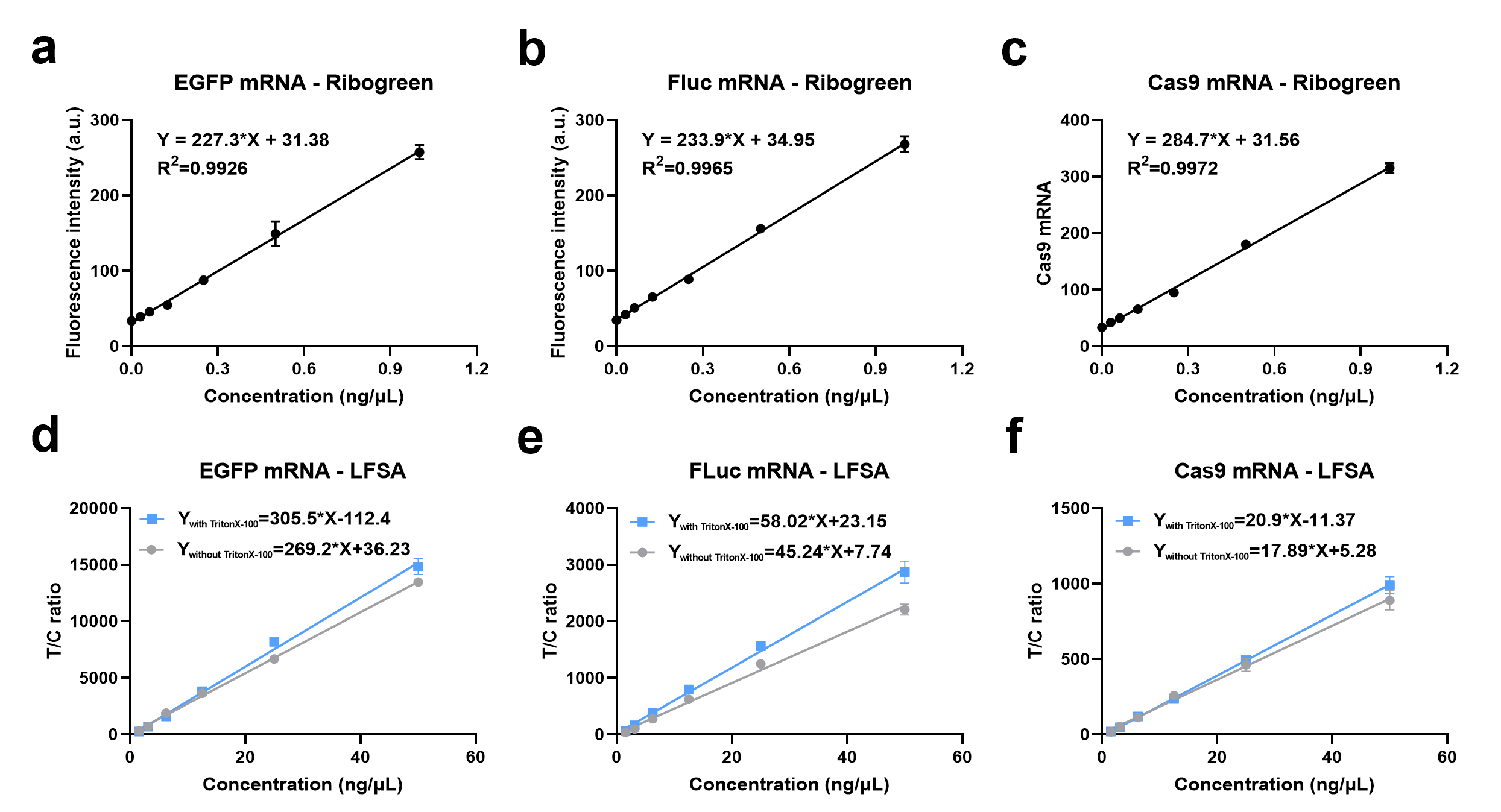
**

**Figure S11. Linear regression analysis of mRNA concentration and fluorescence intensity using the Ribogreen assay and LFSA**

**(a-c)** Linear regression plots between EGFP, Fluc, or Cas9 mRNA concentration and fluorescence intensity using the Ribogreen assay (n = 3).

**(d-f)** Linear regression plots between EGFP, Fluc, or Cas9 mRNA concentration and fluorescence signal of test strips in the presence or absence of 0.2% (v/v) Triton X-100 using the LFSA (n = 3).

**
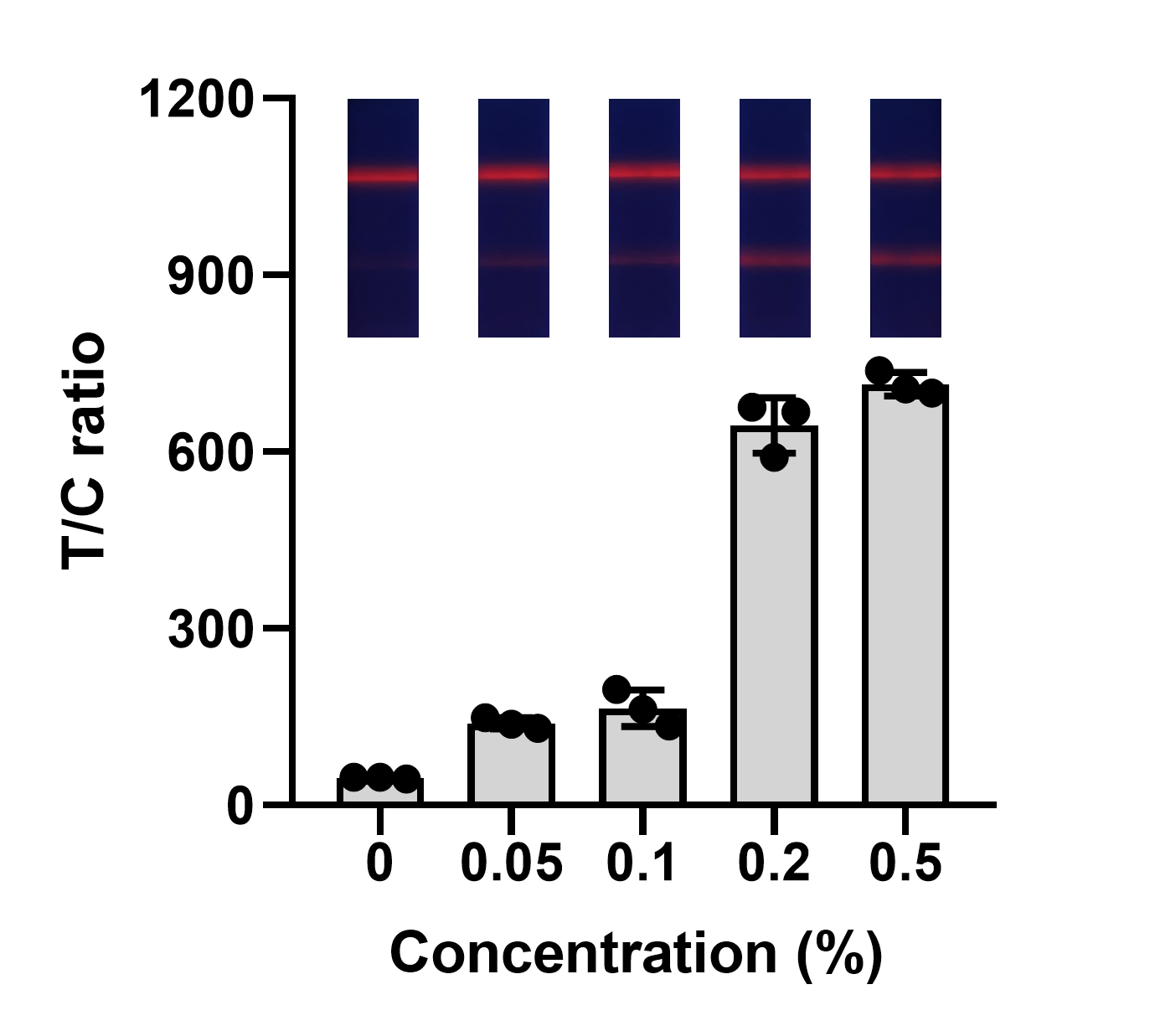
**

**Figure S12. Optimization of Triton X-100 concentration for demulsifying LNPs (n = 3).**

**
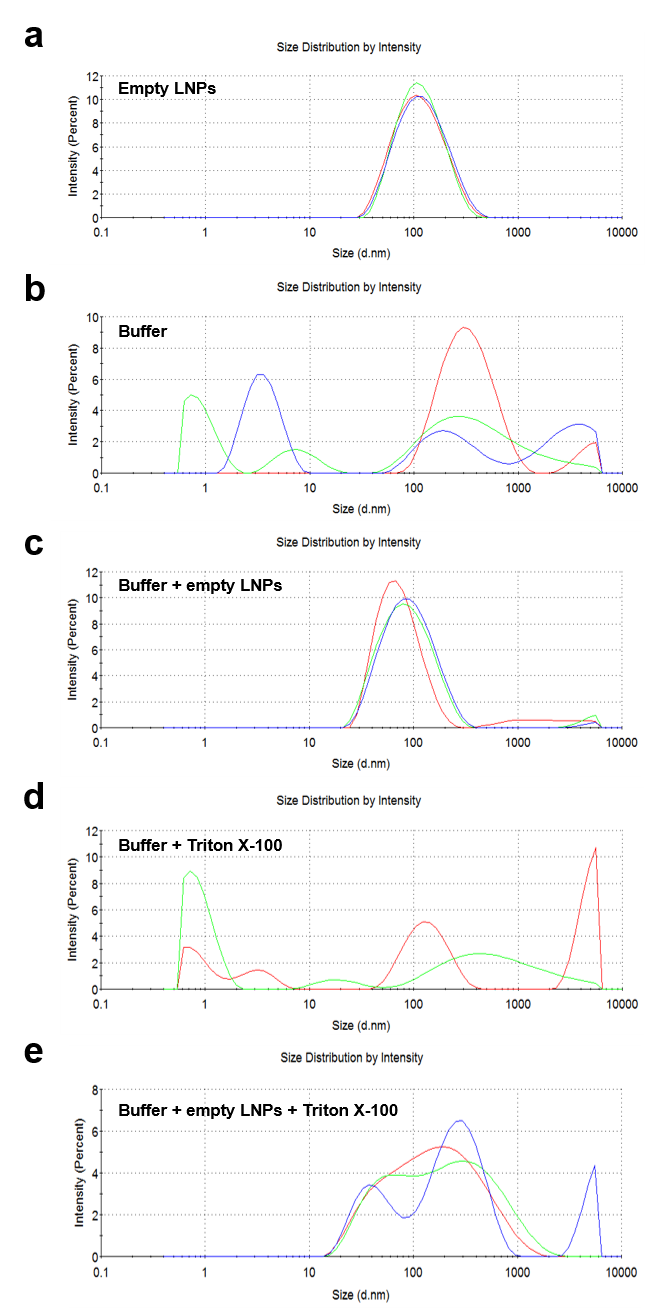
**

**Figure S13. Size distribution by the DLS measurement**

DLS results of empty LNPs **(a)**, buffer **(b)**, buffer + empty LNPs **(c)**, buffer + Triton X-100 **(d)**, and buffer + empty LNPs + Triton X-100 **(e)**.


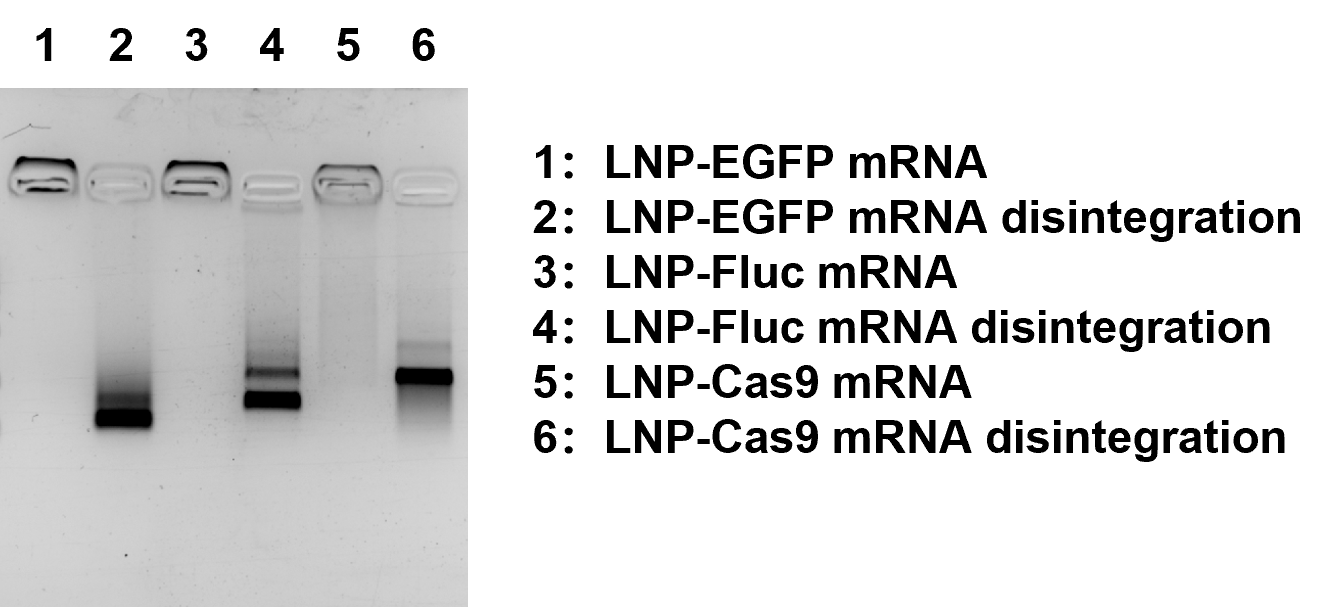


**Figure S14. The encapsulation efficiency of LNP-mRNA was evaluated by 1% agarose gel electrophoresis.**

Lane 1: LNP-EGFP mRNA; Lane 2: LNP-EGFP mRNA with Triton X-100 treatment; Lane 3: LNP-Fluc mRNA; Lane 4: LNP-Fluc mRNA with Triton X-100 treatment; Lane 5: LNP-Cas9 mRNA; Lane 6: LNP-Cas9 mRNA with Triton X-100 treatment.


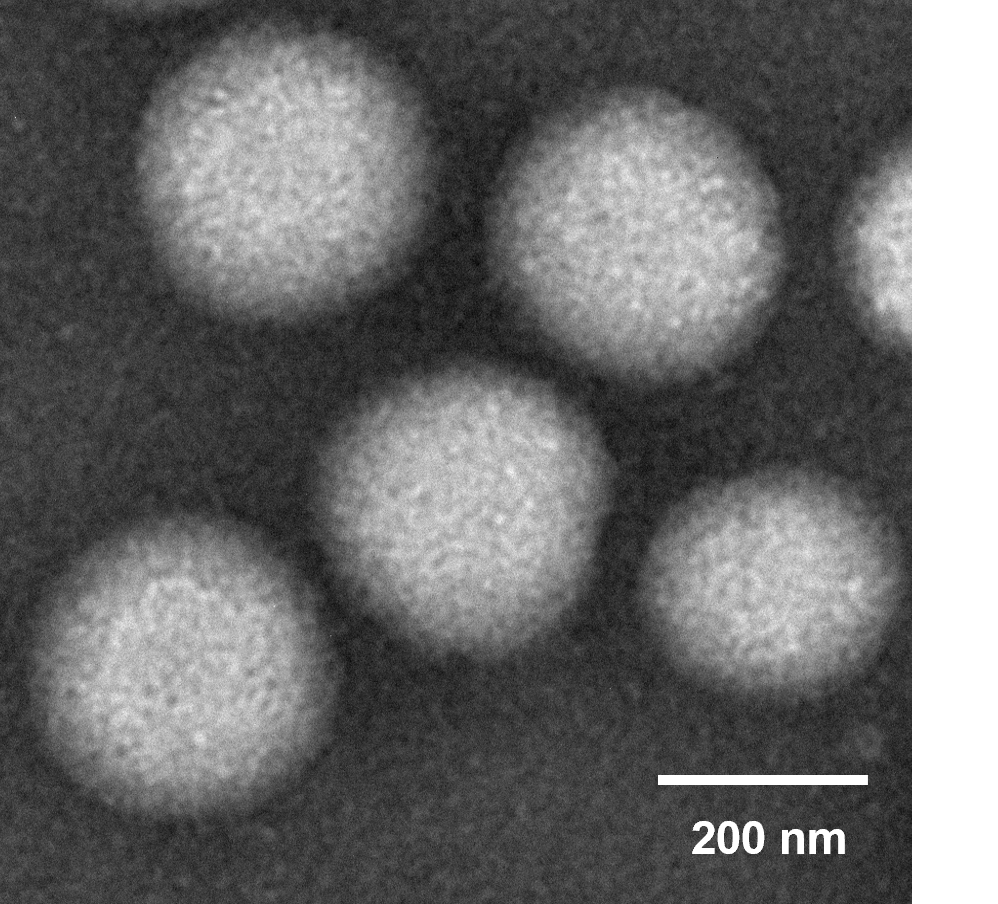


**Figure S15. TEM image of lipid nanoparticles.**


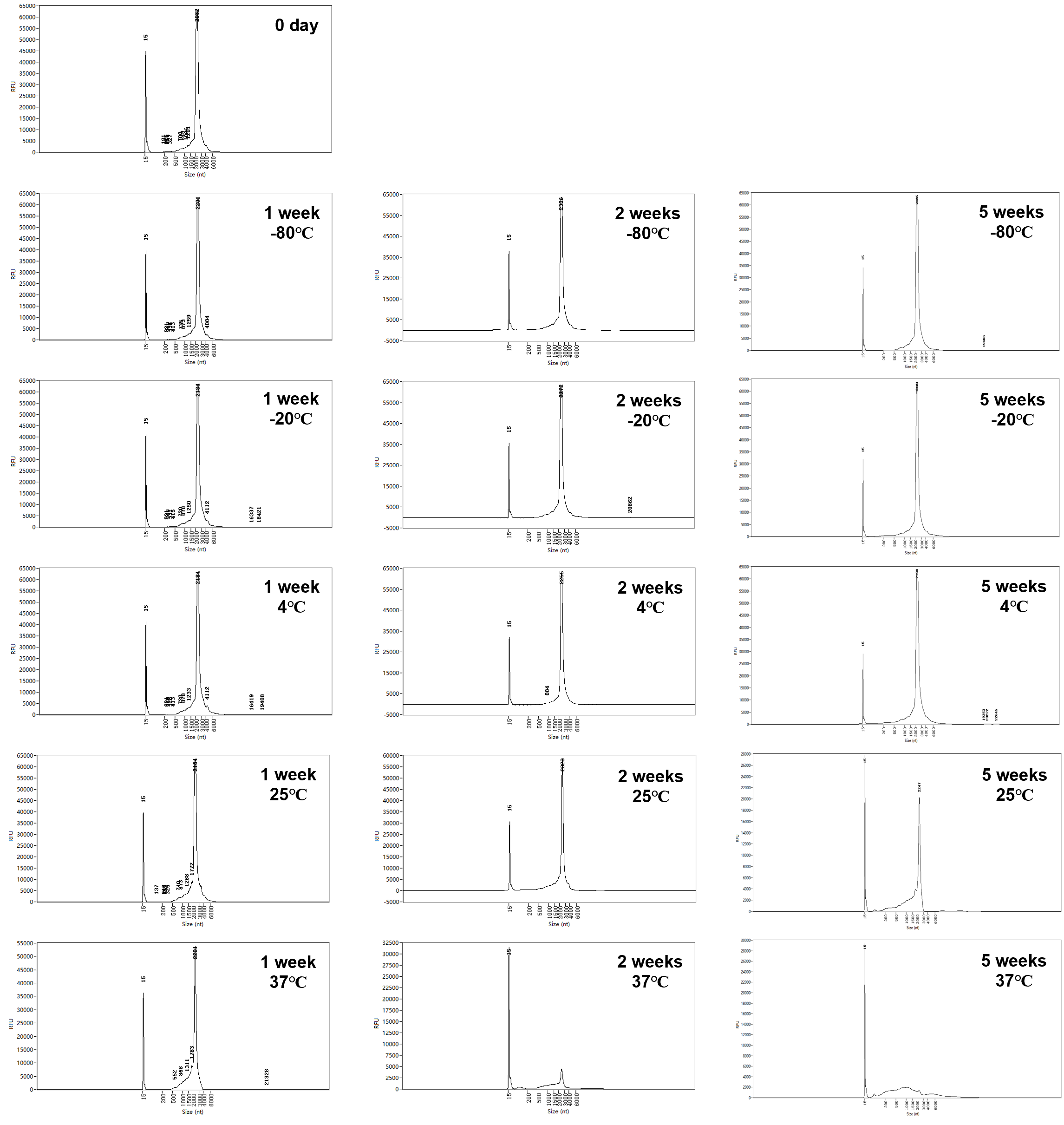


**Figure S16. Electropherograms of Fluc mRNAs in LNPs after storage at -80°C, -20°C, 4°C, 25°C, or 37°C for various durations.**

**
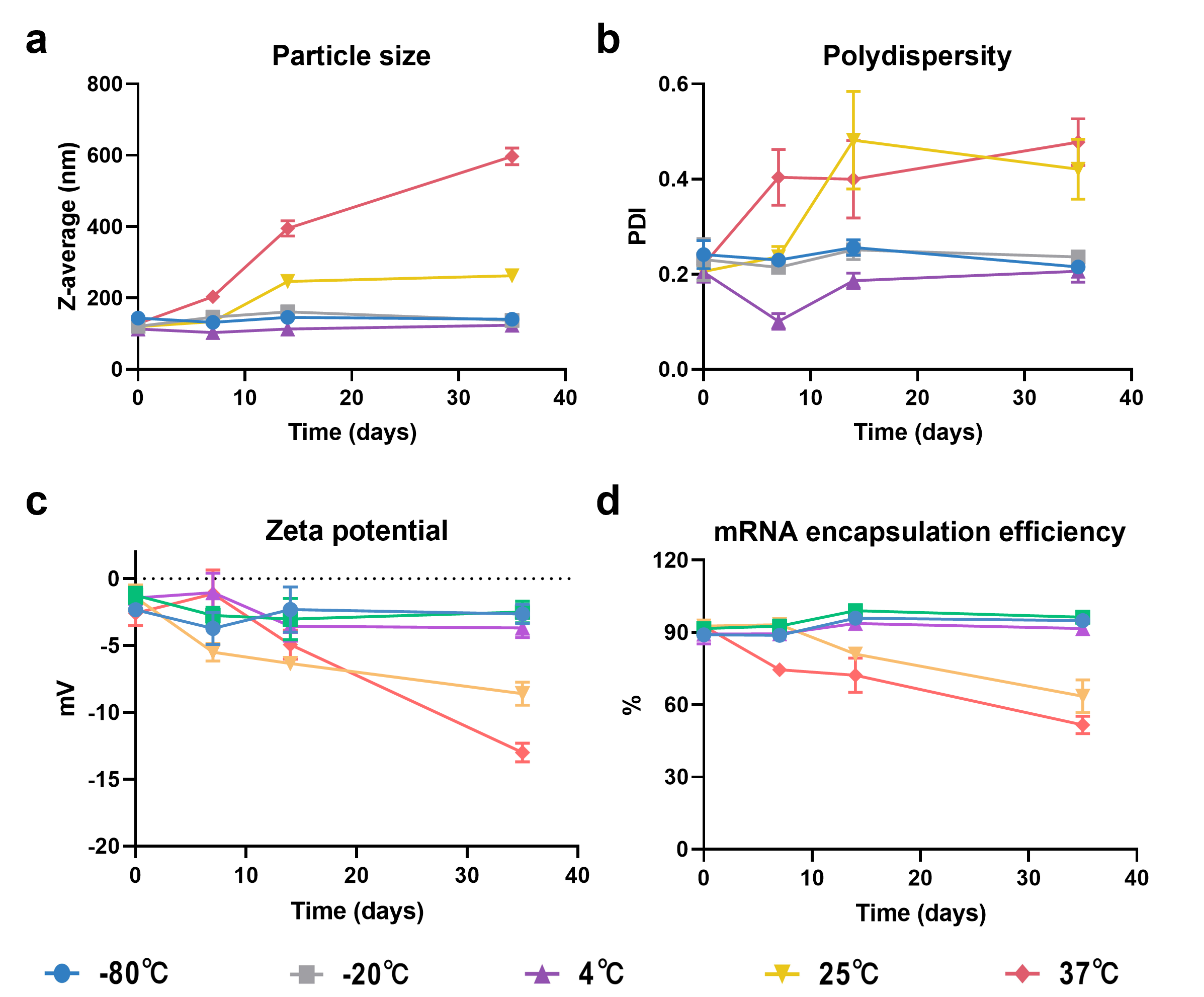
**

**Figure S17. Physicochemical characterization of LNP-mRNA.**

Changes in size **(a)**, PDI **(b)**, zeta potential **(c)**, and encapsulation efficiency **(d)** of LNP-Fluc mRNA after incubation at -80°C, -20°C, 4°C, 25°C, or 37°C for various durations (n = 3).


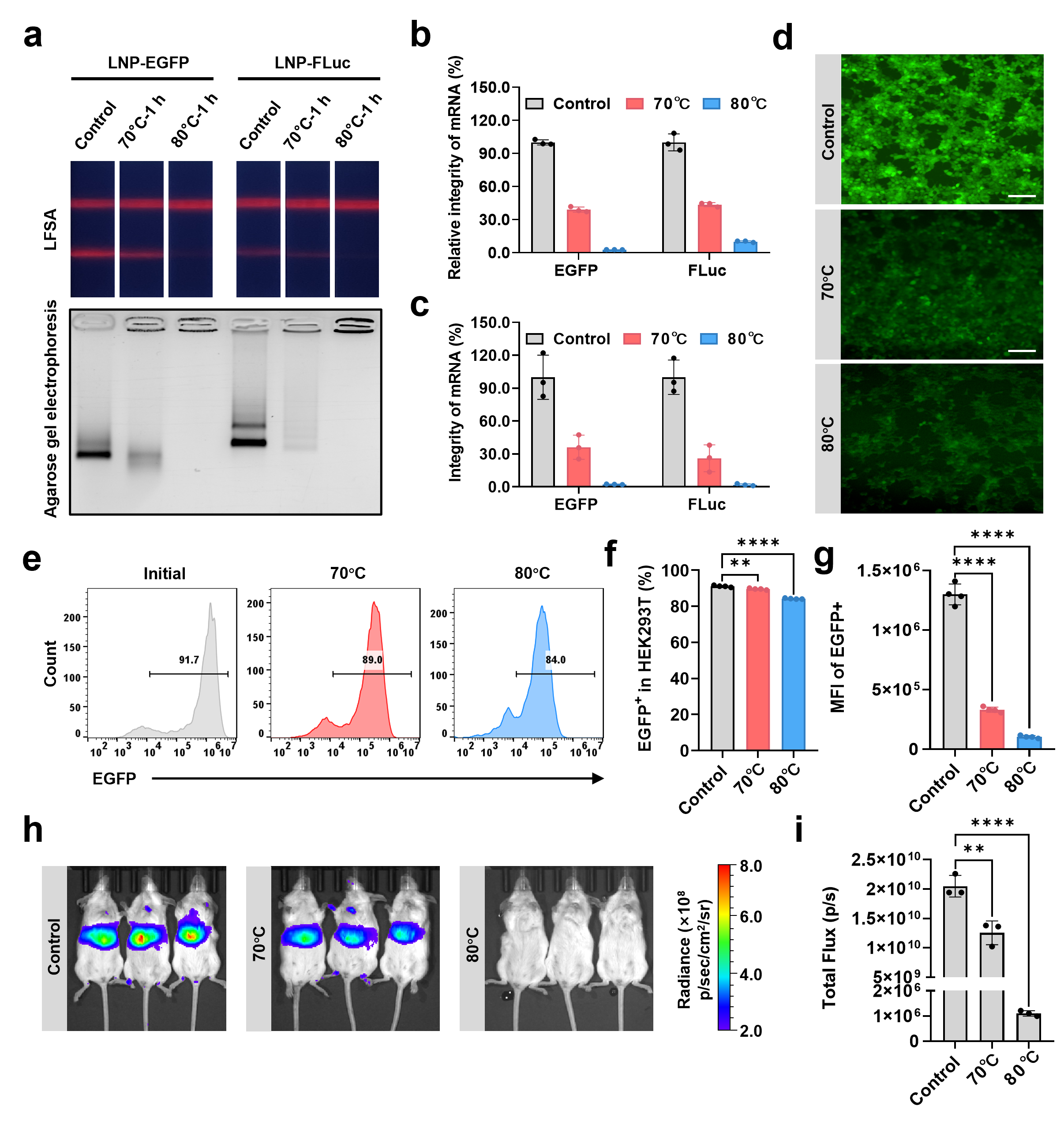


**Figure S18. Validation of LFSA for assessing LNP-mRNA degradation under elevated temperatures via *in vitro* and *in vivo* experiments.**

**(a)** Visual detection using lateral flow strips and agarose gel electrophoresis analysis of LNP-EGFP and Fluc mRNA (100 ng/μL) before and after incubation at 70 °C or 80 °C for 1 h.

**(b)** Quantification of the integrity of mRNA extracted from LNPs based on LFSA using an immunofluorescence analyzer (n = 3).

**(c)** Quantification of the integrity of mRNA extracted from LNPs by gel electrophoresis images analyzed using ImageJ (n = 3).

**(d)** Fluorescence imaging of HEK293T cells after 24 h incubation with LNP-EGFP mRNA under different thermal conditions. Scale bars: 100 μm.

**(e)** Flow cytometry analysis of EGFP expression in HEK293T cells following 24 h transfection with LNP-EGFP mRNA under different thermal conditions.

**(f)** Quantification of transfection efficiency from flow cytometry data in (**e**). Data are presented as mean ± SEM, n=3. *P*-values were calculated using one-way ANOVA with Dunnett's test, ***P*<0.01, *****P*<0.0001.

**(g)** Quantification of mean fluorescence intensity (MFI) of EGFP expression in HEK293T cells from (**e**). Data are presented as mean ± SEM, n=3. *P*-values were calculated using one-way ANOVA with Dunnett's test, *****P*<0.0001.

**(h)** Bioluminescence imaging performed 6 h after intravenous injection of 5 μg LNP-Fluc mRNA, thermally incubated at 70°C or 80°C for 1 h, into BALB/c mice (n = 3 per group).

**(i)** Quantification of bioluminescence signals from (h). Data are presented as mean ± SEM, n=3. *P*-values were calculated using one-way ANOVA with Dunnett's test, ***P*<0.01, *****P*<0.0001.

**
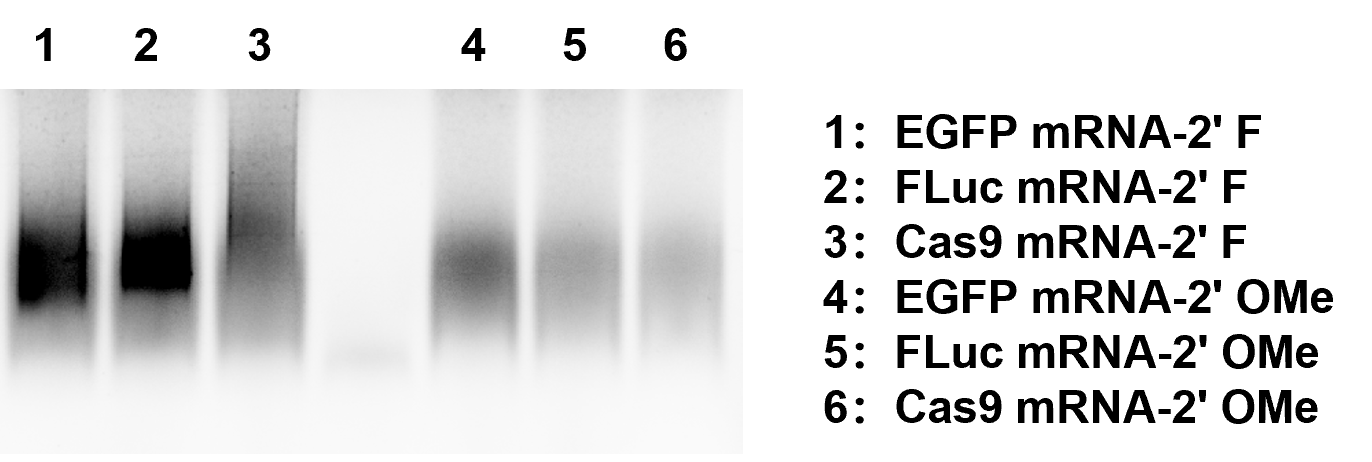
**

**Figure S19. Evaluation of mRNA modified with 2' F or 2' OMe by 1% agarose gel electrophoresis.**

Lane 1: EGFP mRNA modified with 2' F; Lane 2: Fluc mRNA modified with 2' F; Lane 3: Cas9 mRNA modified with 2' F; Lane 4: EGFP mRNA modified with 2' OMe; Lane 5: Cas9 mRNA modified with 2' OMe; Lane 6: Cas9 mRNA modified with 2' OMe.

**Table S1. Probe, cleavage sequence, and cap1 structure for mRNA capping efficiency test.**


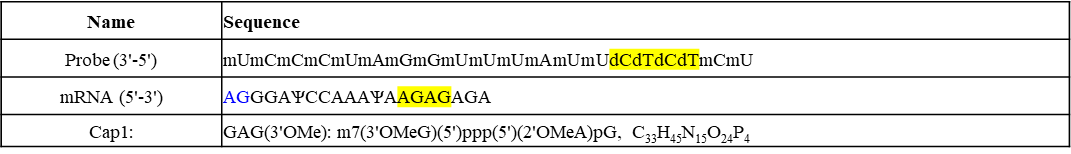


**Table S2. Observed and theoretical cleavage ions for various mRNA cleavage products.**


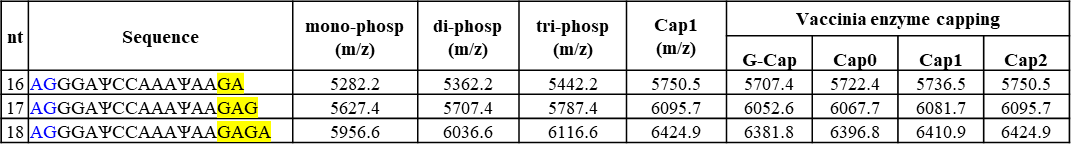


**Table S3. The calculation table of EGFP mRNA capping efficiency.**


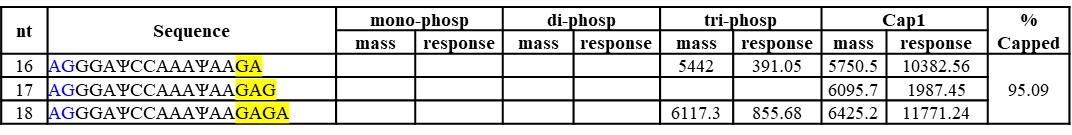


**Table S4. The calculation table of Fluc mRNA capping efficiency.**

**
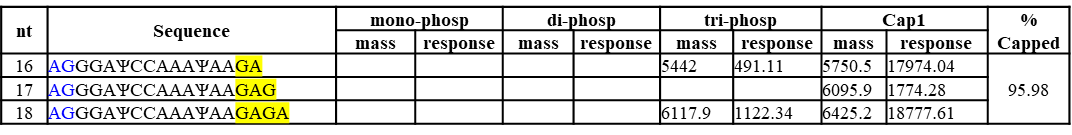
**

**Table S5. The calculation table of Cas9 mRNA capping efficiency.**


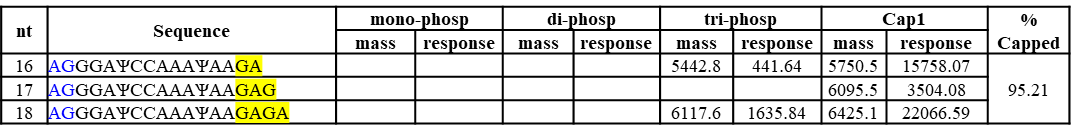


**Table S6. The calculation table of Fluc mRNA (enzyme method) capping efficiency.**


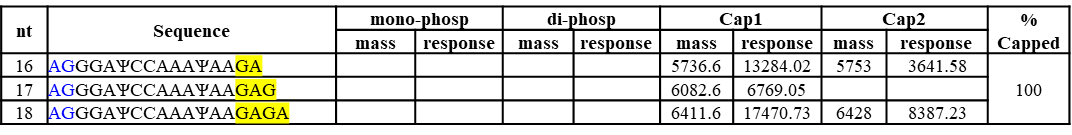


**Table S7. Particle size and PDI results of empty LNPs, buffer, and other combinations.**

| **Sample** | **d. nm** | **PDI** |
| --- | --- | --- |
| Empty LNPs | 100.83 ± 2.62 | 0.17 ± 0.02 |
| Buffer | 142.75 ± 132.78 | 0.60 ± 0.30 |
| Buffer + empty LNPs | 75.14 ± 4.00 | 0.26 ± 0.02 |
| Buffer + Triton X-100 | 300.56 ± 311.32 | 0.53 ± 0.08 |
| Buffer + empty LNPs + Triton X-100 | 134.92 ± 57.86 | 0.49 ± 0.16 |

**Table S8. Comparison of mRNA capping efficiency detection by LC-MS and LFSA.**

| **Quality attribute** | **5' capping efficiency** | |
| --- | --- | --- |
| **Method** | **LC-MS** | **LFSA** |
| **Preparation time** | 130 - 190 min | 90 - 120 min |
| **Assay time** | 30 min | 15 min |
| **Universality** | Design probe for each mRNA | All mRNA |
| **Resolution** | - | 5% |
| **Cost** | High | Low |
| **Sample volume** | 100 pmol (40 - 80 μg) | ≤100 ng |
| **Throughput** | Low | Medium |
| **Data analysis** | Difficulty | Easy |
| **Operation** | Professional | Easy |
| **Experimental environment** | Laboratory | On-site |
| **Equipment** | Precision and costly instrument | Portable device |

**Table S9. Comparison of mRNA integrity detection by capillary electrophoresis and LFSA.**

| **Quality attribute** | **Integrity** | |
| --- | --- | --- |
| **Method** | **Capillary electrophoresis** | **LFSA** |
| **Preparation time** | 10 - 30 min | 5 - 10 min |
| **Assay time** | 60 - 90 min | 15 min |
| **LOD (Fluc mRNA)** | 0.54 ng/μL | 1.94 ng/μL |
| **Linear range** | 1 - 20 ng/μL | 2.5 - 50 ng/μL |
| **Quantification accuracy** | ± 20% | ± 10% |
| **Quantification precision** | 10% CV | ＜10% CV |
| **Cost** | Meidum | Low |
| **Operation** | Professional | Easy |
| **Experimental environment** | Laboratory | On-site |
| **Equipment** | Precision and costly instrument | Portable device |

**Table S10. Comparison of mRNA encapsulation efficiency and concentration detection by Ribogreen assay and LFSA.**

| **Quality attribute** | **Encapsulation efficiency and concentration** | |
| --- | --- | --- |
| **Method** | **Ribogreen assay** | **LFSA** |
| **Preparation time** | 20 - 30 min | 20 - 30 min |
| **Assay time** | 10 min | 15 min |
| **LOD (Fluc mRNA)** | 0.015 ng/μL | 1.94 ng/μL |
| **Linear range (Fluc mRNA)** | 0.03 - 1 ng/μL | 2.5 - 50 ng/μL |
| **Cost** | Low | Low |
| **Sensitive to mRNA degradation** | Low | High |
| **Operation** | Easy | Easy |
| **Experimental environment** | Laboratory | On-site |
| **Equipment** | Precision and costly instrument | Portable device |

**Table S11. The operation procedure and approximate cost of the materials needed for the LFSA assay.**

| **Procedure** | **Materials needed** | **Appropriate cost of materials** |
| --- | --- | --- |
| **Preparation**  **(5' Capping efficiency)** | RNA 5' polyphosphatase & RNA 5' polyphosphatase buffer | US＄4.8 |
|  | Terminator 5' phosphate-dependent exonuclease & Terminator 5' phosphate-dependent exonuclease buffer A | US＄4.3 |
|  | Magnetic beads  Incubator | US＄1.1  US＄300 |
| **Preparation**  **(Integrity)** | Lysis buffer | US＄0.1 |
| **Preparation**  **(Encapsulation efficiency)** | Lysis buffer | US＄0.1 |
| **Testing process** | Sampling buffer | US＄0.1 |
|  | Test card | US＄0.6 |
|  | Fluorescence analysis device | US＄1500 |
| **Total** | US＄10.9 / 5' Capping efficiency test*  US＄0.8 / Integrity test*  US＄0.8 / Encapsulation efficiency test* | |

*Note: The overall cost of each test according to the LFSA test kit, not including the incubator and fluorescence analysis device which can be reused.

**Table S12. Description of DNA primers used in this study.**

| **Name** | **Sequences (from 5′ to 3′)** |
| --- | --- |
| IVT mRNA template Forward | GCCAAGCTTTAATACGACTCACTATAGGGGATCC |
| IVT mRNA template Reverse | TTTTTTTTTTTTTTTTTTTTTTTTTTTTTTTTTTTTTTTTTTTTTTTTTTTTTTTTTTTTTTTTTTTTTTTTTTTTTTTTTTTTTTTTTTTTTTTTTTTTTTTTTTTTTTTTTTTTTTTTCTGCAGGCCGCCCACTCAGACTTTATTC |

**Table S13. Description of mRNA transcripts used in this study.**

| **Description** | **Length (nt)** | **Expected molar mass (Da)** ***** |
| --- | --- | --- |
| CD40 | 771 | 255575 |
| NanoLuc | 816 | 270425 |
| mCherry | 1011 | 334775 |
| mRuby2 | 1014 | 335765 |
| EGFP | 1020 | 337745 |
| CD40L | 1077 | 356555 |
| OVA | 1461 | 483275 |
| Fluc | 1947 | 643655 |
| Cas13a | 3759 | 1241615 |
| Spike | 3921 | 1295075 |
| Cas9 | 4407 | 1455455 |

***Estimated molar mass is calculated by: (nucleotides*330 Da) + 1145 Da for 5**' **cap**
